# PAICS, a Purine Nucleotide Metabolic Enzyme, is Involved in Tumor Growth and the Metastasis of Colorectal Cancer

**DOI:** 10.3390/cancers12040772

**Published:** 2020-03-25

**Authors:** Sumit Agarwal, Balabhadrapatruni V. S. K. Chakravarthi, Michael Behring, Hyung-Gyoon Kim, Darshan S. Chandrashekar, Nirzari Gupta, Prachi Bajpai, Amr Elkholy, Sai A. H. Balasubramanya, Cherlene Hardy, Sameer Al Diffalha, Sooryanarayana Varambally, Upender Manne

**Affiliations:** 1 Department of Pathology, University of Alabama at Birmingham, Birmingham, AL Birmingham, AL 35233, USA; sumitagarwal@uabmc.edu (S.A.); chakravv@gmail.com (B.V.S.K.C.); michaelbehring@uabmc.edu (M.B.); hgkim@uabmc.edu (H.-G.K.); sdarshan@uab.edu (D.S.C.); pbajpai@uabmc.edu (P.B.); dr_amrelkholy@hotmail.com (A.E.); shodigere@gmail.com (S.A.H.B.); saldiffalha@uabmc.edu (S.A.D.); 2 Department of Chemistry, University of Alabama at Birmingham, Birmingham, AL Birmingham, AL 35233, USA; nirzari@uab.edu; 3 Department of Radiation Oncology, University of Alabama at Birmingham, Birmingham, AL Birmingham, AL 35233, USA; hardy@uab.edu; 4 O’Neal Comprehensive Cancer Center, University of Alabama at Birmingham, Birmingham, AL Birmingham, AL 35233, USA

**Keywords:** colorectal cancer, PAICS, epithelial mesenchymal transition, miR-128, metastasis, JQ1

## Abstract

The identification of colorectal cancer (CRC) molecular targets is needed for the development of drugs that improve patient survival. We investigated the functional role of phosphoribosylaminoimidazole carboxylase, phosphoribosylaminoimidazole succinocarboxamide synthetase (PAICS), a de novo purine biosynthetic enzyme involved in DNA synthesis, in CRC progression and metastasis by using cell and animal models. Its clinical utility was assessed in human CRC samples. The expression of PAICS was regulated by miR-128 and transcriptionally activated by Myc in CRC cells. Increased expression of PAICS was involved in proliferation, migration, growth, and invasion of CRC cells irrespective of the p53 and microsatellite status. In mice, the depletion of PAICS in CRC cells led to reduced tumor growth and metastatic cell dissemination to the liver, lungs, and bone. Positron emission tomography imaging showed significantly reduced metastatic lesions in stable PAICS knockdown CRC cells. In cells with PAICS knockdown, there was upregulation of the epithelial mesenchymal transition marker, E-cadherin, and bromodomain inhibitor, JQ1, can target its increased expression by blocking Myc. PAICS was overexpressed in 70% of CRCs, and was associated with poor 5-year survival independent of the pathologic stage, patient’s race, gender, and age. Overall, the findings point to the usefulness of PAICS targeting in the treatment of aggressive colorectal cancer.

## 1. Background

Colorectal cancer (CRC) is the third leading cause of cancer-related deaths in the USA, accounting for 17% of all cancer deaths; it is predicted to cause 51,020 deaths during 2019 [1]. The dysregulation of tumor suppressor genes and oncogenes is involved in the transition from normal colonic epithelium to carcinoma and, eventually, to invasive and metastatic cancer. In CRCs, there is dysregulation of amino acid metabolism, the tricarboxylic acid cycle [2], and synthetic processes for lipids/steroids and nucleotides [3]. Although recent advances in chemotherapy have improved the survival of CRC patients, therapeutic failures are common due to adverse drug effects and the emergence of drug resistance [4]. Therefore, the identification of new targets for therapies and the development of more effective treatments are required to treat CRC patients.

Metabolic alteration is a hallmark of cancer, and there is an association between changes in metabolic genes with cancer cell survival and metastasis [5]. In 35–45% of CRCs, the guanosine triphosphate/guanosine diphosphate GTP/GDP binding protein, ki-ras2 kirsten rat sarcoma viral oncogene homolog (KRAS), is mutated, and this alteration affects treatment strategies [6]. In CRC cells, the malate dehydrogenase 2 inhibitor, compound 7, decreases HIF-α accumulation by reducing oxygen consumption and ATP production and leads to suppressed growth [7]. An enzyme that regulates glycolysis, 6-phosphofructo-2-kinase/fructose-2,6-biphosphatase 3 (PFKFB3), is a target for cancer chemotherapy, for the small-molecule inhibitors of PFKFB3 reduce growth of CRC cells [8].

Purine metabolism is required for the biosynthesis of nucleotides, which are involved in various cellular processes, such as the synthesis of DNA and cofactors such as nicotinamide adenine dinucleotide. In proliferating cells, these processes are upregulated during the G1 and S phases of the cell cycle [9]. Purine nucleotides are formed through the salvage pathway, which utilizes preformed purines and pyrimidines derived from the degradation of cellular nucleic acids, and through the de novo purine biosynthetic pathway. There is a five-fold increase in de novo purine biosynthesis when HCT116 human colon carcinoma cells progress from the mid-G1 to the S phase [10]. Thus, in rapidly dividing cancer cells, the targeting of de novo purine metabolism is a potentially viable strategy to block tumor growth [11]. Phosphoribosylaminoimidazole carboxylase, phosphoribosylaminoimidazole succinocarboxamide synthetase (PAICS), a de novo pathway metabolic enzyme, produces an intermediary metabolite, N-succinyl-5-aminoimidazole-4-carboxamide-1-ribose-5’-phosphate (SAICAR), and, under glucose-depleted stress conditions, activates pyruvate kinase isoform pyruvate kinase muscle isozyme M2 (PKM2) [12]. The interaction of SAICAR with PKM2 promotes the survival of cancer cells [12]. For human carcinomas, inhibition of aminoimidazole carboxamide ribonucleotide transformylase (ATIC), which catalyzes the last two steps of de novo purine biosynthesis and decreases cell proliferation and cell cycle progression, highlighting a potential role for ATIC inhibitors in cancer therapy [13].

Our previous studies have demonstrated a function for PAICS in cancer cell proliferation and the invasion and growth of lung [14], prostate [15], and bladder cancers [16]. Furthermore, PAICS is essential for breast cancer growth [17,18]. However, there are no reports regarding the role of PAICS in CRC. Moreover, its role in metastasis has not been studied in any human malignancy. Therefore, in the present study, we characterized the expression of PAICS in CRCs and investigated the association of PAICS with CRC progression and metastasis. Our analyses showed increased PAICS expression in CRCs compared to matched normal colonic epithelial tissues. We also established that PAICS was involved in cellular growth, colony formation, invasion, and the spheroid-forming capacity of CRC cells as well as in tumor growth and metastasis in animals. Furthermore, microRNA (miR)-128 negatively regulated PAICS expression. In CRCs, miRNA-128 is downregulated [19], and its downregulation results in the overexpression of PAICS. Increased levels of E-cadherin after PAICS knockdown indicate that the epithelial-mesenchymal transition (EMT) is modulated through PAICS. Furthermore, the present study shows that treatment of CRC cells with JQ1, a bromodomain inhibitor, reduces PAICS expression. Thus, the results reveal that PAICS is a promising target for treatment of CRC.

## 2. Results

### 2.1. PAICS is Overexpressed in CRCs

**Figure 1:**
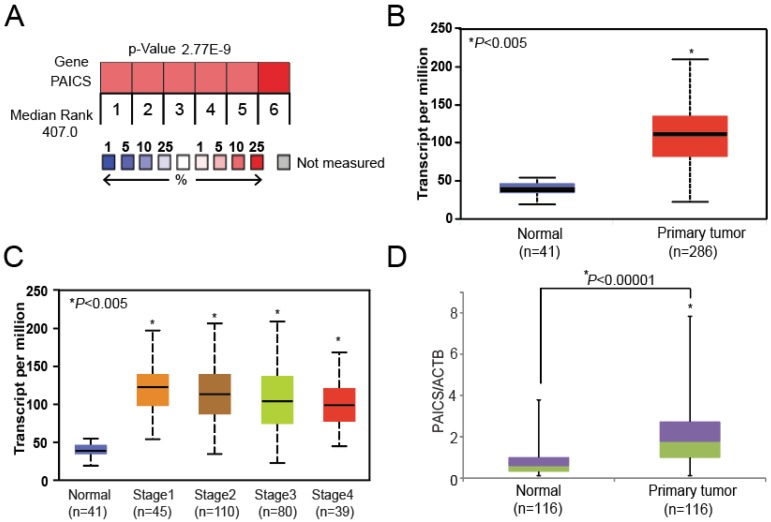

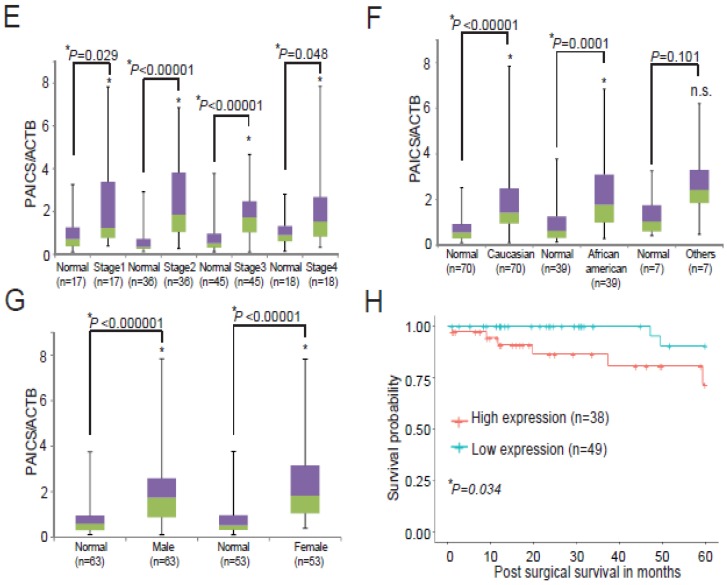
PAICS mRNA overexpression in colorectal cancers (CRCs). (**A**) Information on gene expression profiling obtained from the Oncomine database [20] showing the expression of phosphoribosylaminoimidazole carboxylase, phosphoribosylaminoimidazole succinocarboxamide synthetase PAICS in CRC tissues. (**B**) UALCAN data [22] showing greater PAICS expression in colon adenocarcinomas (*n* = 286) compared to normal colon tissues (*n* = 41). (**C**) UALCAN data showing stage-wise PAICS expression. (**D**) In-house validation by quantitative polymerase chain reaction (qPCR) showing PAICS expression using RNA from paired CRC (*n* = 116) and normal colon tissues (*n* = 116). qPCR analysis showing PAICS expression in various pathologic stages (**E**). Race (**F**), and gender (**G**). For qPCR normalization, β-actin (ACTB) was used as a reference gene. (**H**) Kaplan-Meier survival curves based on the PAICS mRNA expression status of CRC patients (blue lines indicate patients with low PAICS expression (*n* = 49); red lines indicate patients with high (*n* = 38) PAICS expression).

With data on the expression of CRC genes available in the Oncomine database [Oncomine™ Platform (Life Technologies, Ann Arbor, MI, USA)] [20], we found increases in PAICS expression in several independent profiling studies (Figure 1A). Such studies acquired from Skrzypczak datasets [21] available in Oncomine, showed, for CRCs, increased expression of the de novo purine biosynthetic enzymes, ATIC, glycinamide ribonucleotide synthetase/glycinamide ribonucleotide transformylase/aminoimidazole ribonucleotide synthetase (GART), phosphoribosyl pyrophosphate amidotransferase (PPAT), adenylosuccinate lyase (ADSL), phosphoribosylformylglycinamidine synthase (PFAS), and PAICS (Supplementary Figure S1). The publicly available UALCAN bioinformatics website (http://ualcan.path.uab.edu) [22] provided TCGA data showing that PAICS was over-expressed in colon adenocarcinomas compared to normal colon tissues (Figure 1B). Stage-wise expression provided by UALCAN showed that PAICS was overexpressed across all stages compared to normal tissues and that expression was independent of the pathological stage (Figure 1C). In addition, PAICS upregulation was independent of race (Figure S2A), gender (Figure S2B), age (Figure S2C), and weight (Figure S2D). Using qPCR analysis, the increased expression of PAICS mRNA was validated in CRC tissues (*n* = 116) relative to adjacent paired normal colorectal tissues (*n* = 116) (Figure 1D). qPCR analysis confirmed PAICS upregulation in Stage 1 (*n* = 17), Stage 2 (*n* = 36), Stage 3 (*n* = 45), and Stage 4 (*n* = 18) of CRCs compared to paired normal tissues (Figure 1E). PAICS expression was also independent of race (Caucasian (*n* = 70) vs. African-American (*n* = 39)) (Figure 1F) (Table S1), gender (male (*n* = 63) vs. female (*n* = 53)) (Figure 1G), and age (21–40 years (*n* = 7), 41–60 years (*n* = 36), 61–80 years (*n* = 57), and 81–100 years (*n* = 16)) (Figure S2E). These results established that PAICS mRNA was overexpressed in CRCs as compared to their corresponding normal tissues, irrespective of the stage of progression, race, gender, and age.

### 2.2. PAICS Overexpression is Associated with Poor Survival

Time to event analysis in a subset of patients with qPCR data (*n* = 87) found that PAICS upregulation is significantly associated with poor 5-year survival (log-rank *p*-value = 0.034, Figure 1H). After adjusting for stage, the hazard of death from cancer in patients with high PAICS expression was 4.7 times higher than in patients with low PAICS expression (95% CI 0.95–23.39), *p*-value = 0.058, Table S3).

### 2.3. Upregulation of PAICS Protein in CRCs

**Figure 2:**
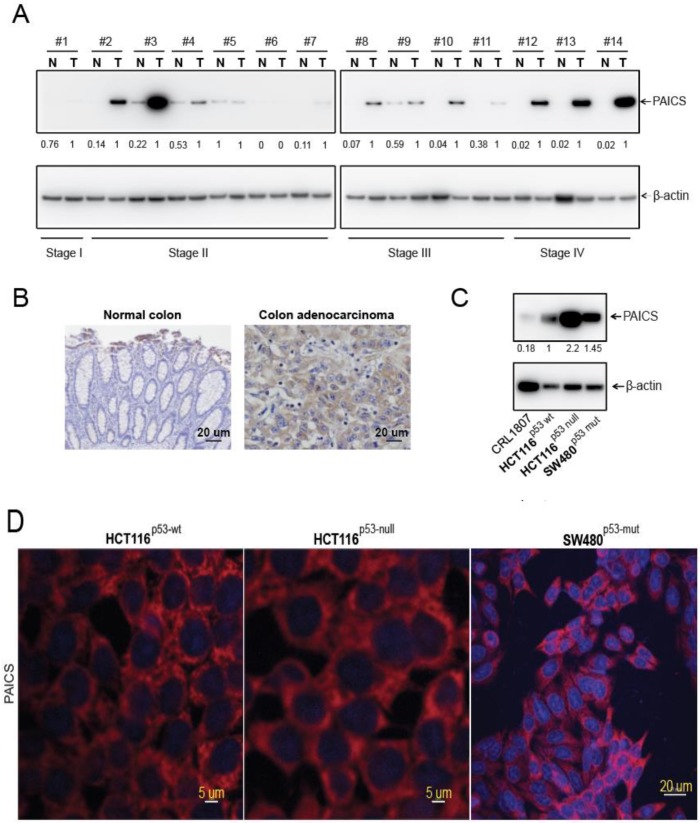
PAICS protein upregulation in CRCs. (**A**) Immunoblot analysis showing stage-wise expression of PAICS in paired CRCs and corresponding normal colorectal tissues. β-Actin was used as a loading control. Densitometric analysis was performed individually for each sample (#1–14) having normal (N) and tumor (T). The numerical values indicate the normalized intensity ratio of PAICS/β-actin. (**B**) Representative images of immunohistochemical (IHC) analysis of CRC tissue sections showing PAICS reactivity in CRCs compared with normal colorectal tissue. Hematoxylin was used for nuclear staining; scale bar—20 µm (**C**) Immunoblot analysis showing PAICS expression across a panel of CRC cell lines. β-Actin was used as a loading control. (**D**) Immunofluorescence analysis showing PAICS cytoplasmic localization in HCT116^p53-wt^ and HCT116^p53-null^ cells; scale bar—5 µm and SW480^p53-mut^; scale bar—20 µm.

After confirming PAICS expression in CRC tissues, we investigated PAICS protein expression by immunoblot analysis. Our analyses show higher expression in all pathological stages of CRC tissues compared with matched normal colorectal tissues (Figure 2A). We further validated PAICS protein expression by employing the immunohistochemical (IHC) analysis of normal and CRC tissues. There was strong cytoplasmic staining in CRC tissue sections as compared to normal sections (Figure 2B). By using immunoblot analyses, we also confirmed PAICS protein expression in CRC cells (HCT116^p53-wt^, HCT116^p53-null^, and SW480^p53-mut^), relative to CRL1807 SV-40 transformed colon cells (Figure 2C). The immunofluorescence analysis revealed that PAICS protein expression was predominantly localized in the cytoplasm of HCT116^p53-wt^, HCT116^p53-null^, and SW480^p53-mut^ cells (Figure 2D) and HT29^p53-mut^ (Figure S2F). These results demonstrate that CRCs overexpress the PAICS protein irrespective of the disease stage.

### 2.4. Function of PAICS on Growth of CRCs

**Figure 3:**
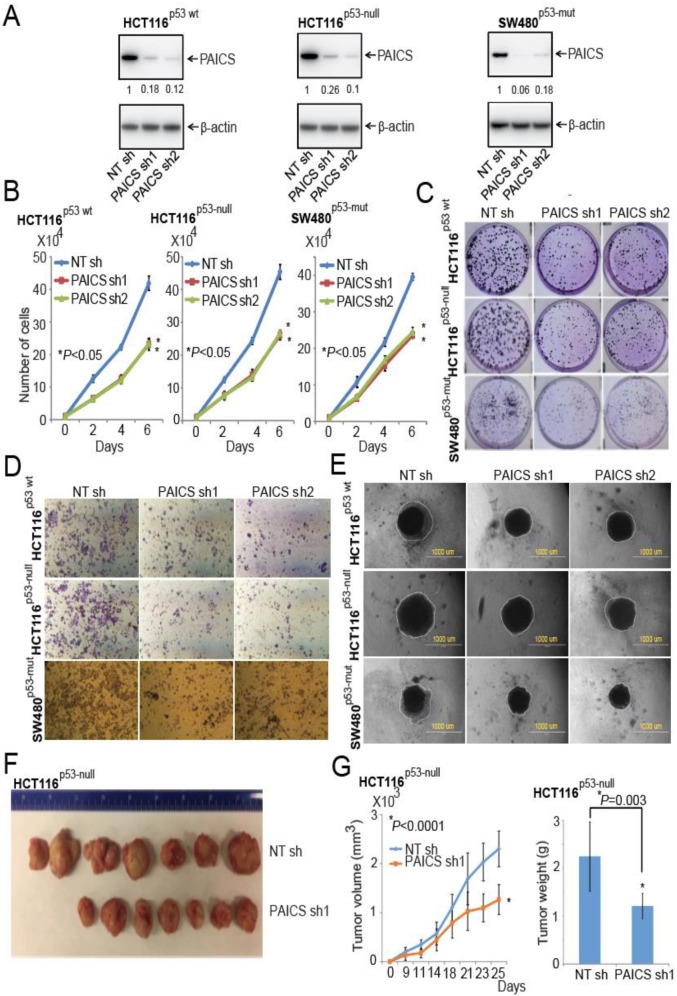
PAICS silencing retards CRC cell and tumor growth. (**A**) Immunoblot analysis using protein lysates from CRC cells, HCT116^p53-wt^, HCT116^p53-null^, and SW480^p53-mut^, which were stably transfected with PAICS shRNAs, showed PAICS protein reduction as compared to cells transfected with NT shRNA. β-Actin was used as a loading control. Densitometric readings for Western Blots were calculated, and values were normalized relative to β-actin. These values are provided below the blots of the PAICS expression. The stable knockdown of PAICS in CRC cells reduced cell proliferation (**B**) and colony formation (**C**). (**D**) Representative images showing less invasion of PAICS-knockdown CRC cells through Transwell Matrigel membranes compared to cells treated with NT shRNA. (**E**) Phase-contrast microscopy images showing reduced formation of spheroids by CRC cells with PAICS knockdown compared with those transfected with NT shRNA; scale bar—1000 µm. (**F**) HCT116^p53-null^ cells transfected with PAICS shRNA1 or control NT shRNA were injected into NSG mice, and tumors were monitored at the indicated time points. A representative photograph showing tumors of two groups: those formed from cells transfected with control NT shRNA (*n* = 7) and those transfected with PAICS shRNA1 (*n* = 7). (**G**) A histogram showing tumor volumes and the weights of mouse xenografts (*n* = 7) +/- SD.

To understand the function of PAICS in the proliferation of CRC cells, we performed cell growth assays. Lentivirus-mediated gene silencing was used to knock down PAICS in the CRC cells HCT116^p53-wt^, an HCT116 cell line with the p53 gene homozygosity disrupted (HCT116^p53-null^), and SW480^p53-mut^ (p53-mutated) and stable cell lines were developed after lentivirus infection by puromycin cell-selection. The immunoblot analysis showed reductions in PAICS protein with PAICS shRNA1 or PAICS shRNA2 (Figure 3A). Furthermore, experiments involving cell proliferation, colony formation, invasion, cell migration, and spheroid formation were performed. Cell proliferation studies revealed reductions in cell counts by 39% (*p* < 0.01) and 37% (*p* < 0.01) with PAICS shRNA1 and shRNA2, respectively, in HCT116^p53-wt^ cells at 6 days as compared to non-targeting (NT) shRNA cells. HCT116^p53-null^ cells showed reductions with PAICS shRNA1 (45%; *p* < 0.01) and PAICS shRNA2 (38%; *p* < 0.01), as did SW480^p53-mut^ cells with PAICS shRNA1 (33%; *p* < 0.01) and PAICS shRNA2 (31%; *p* < 0.01) (Figure 3B). 

PAICS knockdown resulted in a significant reduction (*p* < 0.01) in the colony-forming capacity of HCT116^p53-wt^, HCT116^p53-null^, and SW480^p53-mut^ cells; the reduction was independent of p53 and microsatellite status (Figure 3C; Figure S3A). Transwell membrane assays revealed the effect of the reductions of PAICS on the invasive properties of HCT116^p53-wt^, HCT116^p53-null^, and SW480^p53-mut^ cells (Figure 3D; Figure S3B). We also performed assays involving 3D spheroid cultures, which mimic the features of tumors, such as cell–cell interaction, hypoxia, and deposition of extracellular matrix [23]. For all three cell lines (HCT116^p53-wt^, HCT116^p53-null^, and SW480^p53-mut^), PAICS knockdown reduced their capacity significantly (*p* < 0.05) to form spheroids (Figure 3E; Figure S3C).

Having established the function of PAICS in various malignant properties of cells, we tested CRC cells in a wound-healing assay, which simulates tumor cell motility in animals. Wounds were created in the confluent monolayers of cells transfected with NC shRNA, PAICS shRNA1, or PAICS shRNA2, and images were acquired at 0 and 24 hours. The images showed reduced motility of CRC cells transfected with PAICS shRNA1 or shRNA2 relative to NC shRNA (Figure S4). These data conclude that the reduction in CRC cell proliferation, invasion, cell motility, and spheroid formation after PAICS knockdown is not dependent on the status of p53 and microsatellite.

The effect of PAICS on tumor cell growth was investigated by use of a Chick Chorioallantoic Membrane (CAM) assay. HCT116^p53-null^ cells with stable PAICS knockdown showed that tumor weights were lowered by 46% with PAICS shRNA1 and 36% with PAICS shRNA2 compared with the NT shRNA controls (Figure S5A). To investigate PAICS function in a CRC xenograft model, NOD/SCID/IL2γ-receptor null (NSG) mice were used. Tumors formed by HCT116^p53-null^ cells treated with PAICS shRNAs were smaller compared to those produced by cells treated with a control shRNA (Figure 3F). Upon PAICS knockdown, there were also less tumor growth and lower tumor weights. Tumor volumes were decreased by 44% (*p* = 0.0001) and tumor weights by 46% (*p* = 0.003) (Figure 3G). Thus, these knockdown studies showed PAICS involvement in the malignant properties of CRCs.

### 2.5. PAICS Contributes to CRC Metastasis to Lung, Liver, and Bone

**Figure 4:**
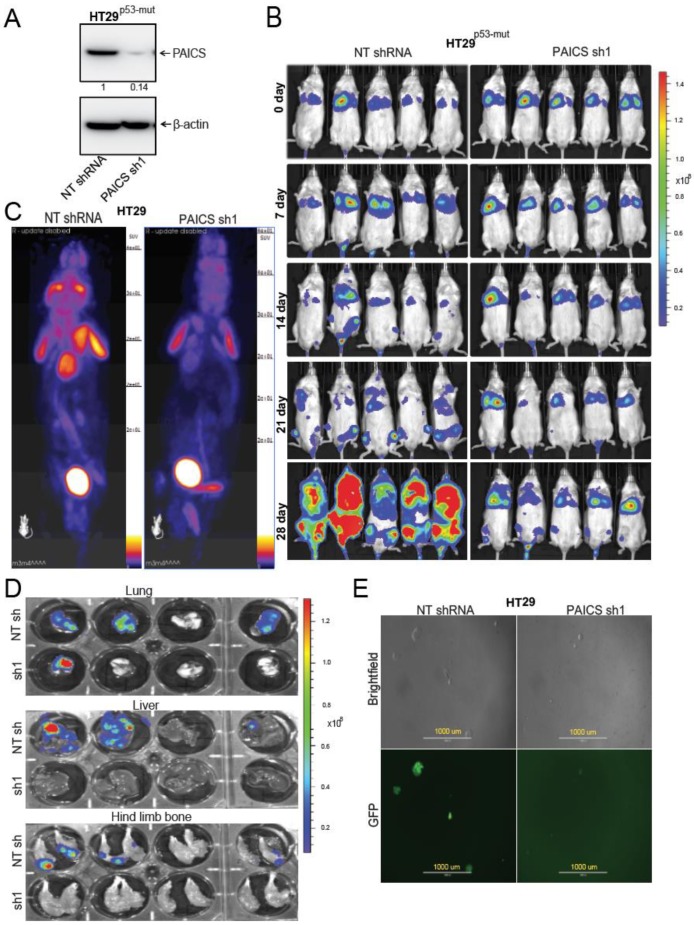
PAICS knockdown inhibits CRC metastasis. (**A**) Immunoblot analysis to show PAICS knockdown efficiency in aggressive HT29^p53-mut^ cells. Densitometric analysis was performed to confirm that the knockdown efficiency was calculated and normalized relative to the β-Actin. (**B**) Representative images of bioluminescence imaging over the course of 4 weeks showing development of metastases in NSG mice after lateral tail-vein injections of tumor cells transfected with NT shRNA (*n* = 5) or PAICS shRNA (*n* = 5). The scale bar showed luminescence in units of radiance (p/sec/cm^2^/sr). (**C**) PET imaging of these mice showing metastatic lesions. The brain, heart, kidney, and bladder show an uptake of fluorodeoxyglucose (FDG). (**D**) The ex vivo luminescence of the liver, hind-limb bone, and lungs of mice injected with control cells or with cells transfected with PAICS shRNA. The scale bar showed luminescence in units of radiance (p/sec/cm^2^/sr). (**E**) Flushed bone marrow cells cultured with media containing puromycin. Phase contrast and green fluorescent protein (GFP) images showing cancer cells procured from bone marrow of mice injected with tumor cells transfected with NT shRNA or with PAICS shRNA; 1000 µm.

For metastasis experiments, HT29^p53-mut^ cells labeled with a luciferase construct were transfected with or without PAICS shRNA followed by selection with puromycin. Stable cell lines were developed and Western Blot analysis was performed to check the knockdown efficiency of PAICS (Figure 4A). After knockdown confirmation of PAICS in HT29^p53-mut^ cells, lateral tail-vein injections were performed in NSG mice. The metastatic progression of the cancer cells was followed for 4 weeks using an IVIS bioluminescence imaging system. Both control and PAICS knockdown cells were found to be present in the lungs. The luminescence data showed that HT29^p53-mut^ cells metastasized to bone via arterial circulation. In all mice injected with tumor cells transfected with NT shRNA, the luciferase signal was evident at 21 days after injection as compared to those injected with tumor cells transfected with PAICS shRNA. At 28 days, for mice injected with PAICS knockdown cells, there was a reduction in luminescence intensity, suggesting a decreased dissemination of tumor cells to liver and bone as compared to mice injected with cells transfected with control shRNA (Figure 4B). For the cells transfected with control shRNA, there were more metastases in the vertebra area near the thorax relative to those formed from cells treated with PAICS shRNA (Figure S5B). Furthermore, fluoro-deoxyglucose-positron emission tomography (FDG-PET) imaging confirmed the ex vivo analysis, showing metastatic lesions in the shoulder area for cells exposed to control NT shRNA (Figure 4C). High luminescence intensity (Figure S5C) and H&E staining (Figure S5D) confirmed that the lesions were tumors. The ex vivo tests showed less luminescence intensity in the liver, lungs, and hind-limb bones of mice injected with PAICS knockdown cells (Figure 4D). As described by Gutierrez-Uzquiza and colleagues [24], we flushed out bone marrow cells from bones and cultured them in puromycin-containing media to select cancer cells. After 5 days of culture, there were lower numbers of cancer cells in cultures from the bone marrow of mice injected with PAICS knockdown cells as compared to cells transfected with NT shRNA (Figure 4E). This data indicated the role of PAICS in CRC metastasis.

### 2.6. Validation of CRC Metastasis to Liver and Bone by Histopathology

**Figure 5:**
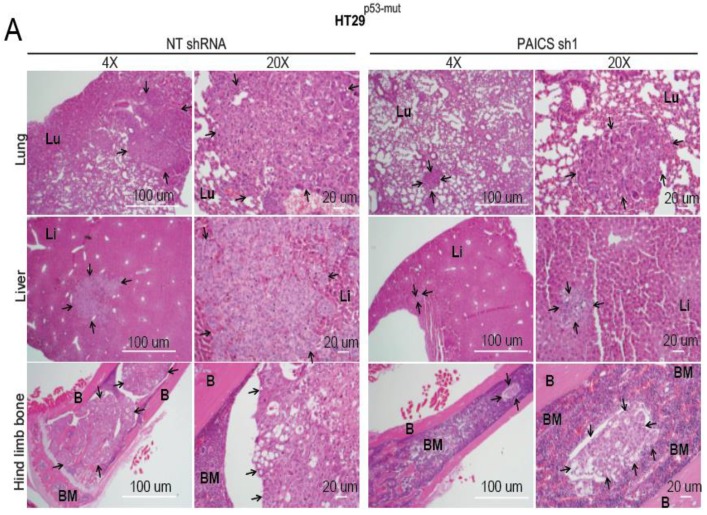

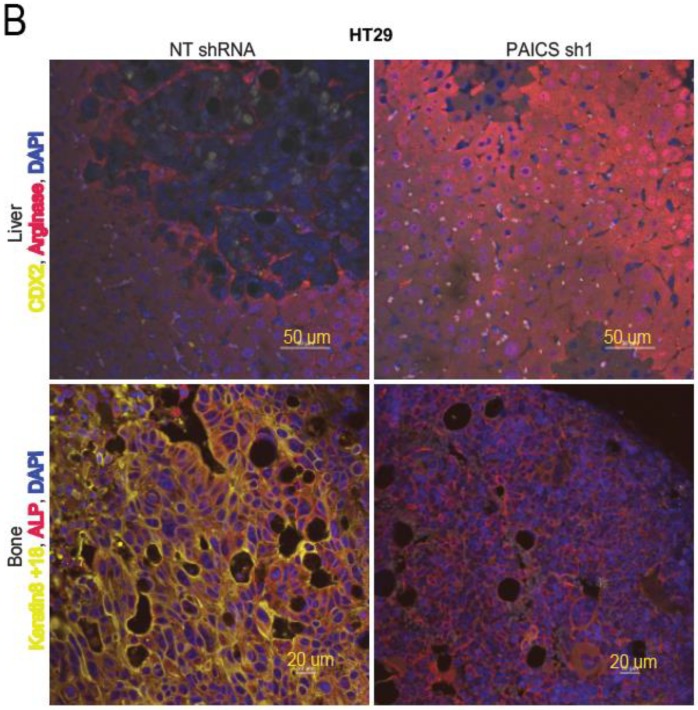
PAICS knockdown decreases the dissemination of CRC lesions to various organs. (**A**) Hematoxylin & eosin (H&E) staining of the hind-limb bones, lungs, and livers of mice injected with cells transfected with NT shRNA or PAICS shRNA. The images were taken at 4×and 20×; scale bar—20 µm. The arrows show metastatic lesions; BM, bone marrow; B, bone; Lu, lungs; Li, liver. (**B**) Immunofluorescence (IF) co-staining of liver with CDX2 as a marker of cancer cells (yellow) and arginase as a marker for liver cells; scale bar—50 µm. For bone staining, keratin 8 + 18 was used as a marker of cancer cells (yellow), ALP as a marker of osteoclast lineage (red), and DAPI for nuclear staining (blue); scale bar—20 µm.

H&E staining of excised organs showed fewer metastatic lesions in the bone marrow, liver, and lungs of those injected with PAICS knockdown cells (Figure 5A). Examination of the ultrastructure of bone by H&E stains showed low amounts of bone marrow and poorly differentiated metastatic lesions in those exposed to NT shRNA, which led to osteolytic metastasis in mice (Figure 5A). We further conducted immunofluorescence staining of the liver and bones with cell-type markers. This was accomplished to validate liver and bone colonization of CRC cells and to show the interaction of CRC cells with liver as well as bone cells [25]. For liver metastasis, we used CDX2 as a marker of cancer cells and arginase as a hepatocyte marker, which showed CDX2 staining surrounded by arginase in control NT shRNA mice (Figure 5B). For bone staining, we used keratin 8 + 18, a marker for cancer cells, and alkaline phosphatase (ALP), a marker for bone marrow mesenchymal stem cells. We found that the bone marrow niche cells expressed the ALP marker in abundance in mice injected with PAICS-depleted cells; those cells exposed to control NT shRNA showed abundant keratin 8 and less ALP surrounding the metastatic lesions (Figure 5B). These animal experiments demonstrated a role of PAICS in CRC metastasis to lung, liver, and bone. 

### 2.7. PAICS Modulates the EMT in CRC Cells

**Figure 6:**
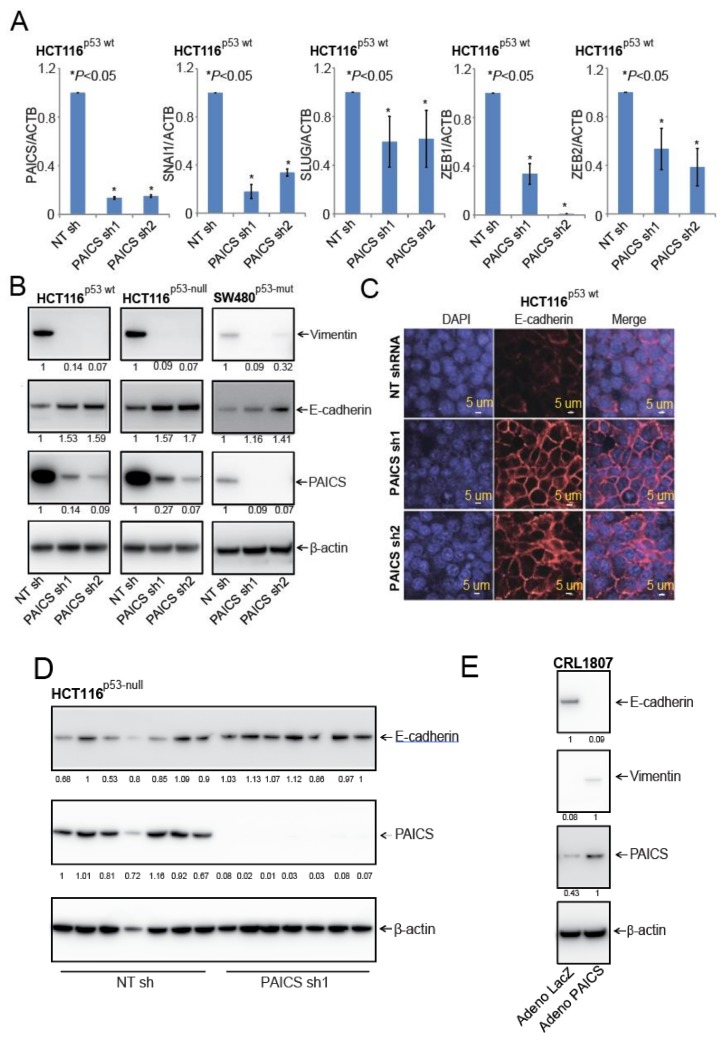
PAICS initiates the epithelial mesenchymal transition (EMT) in CRC. (**A**) qRT-PCR analysis to evaluate mRNA expressions of *PAICS*, *SNAI1*, *SLUG*, *ZEB1*, and *ZEB2* in HCT116^p53-wt^ cells after PAICS knockdown. (**B**) The lysates of PAICS knockdown cells were blotted with antibodies for E-cadherin, vimentin, PAICS, or β-actin. The densitometric readings were calculated and normalized relative to the β-Actin. (**C**) Immunofluorescence for E-cadherin (red fluorescence) and DAPI (blue fluorescence) for nuclear stain in HCT116^p53-wt^ cells with PAICS knockdown; scale bar—5 µm. (**D**) Immunoblot analysis for the expression of PAICS and E-cadherin in tumors excised from control mice or tumors treated with PAICS shRNA. The normalized ratio of the PAICS/β-Actin is reported. (**E**) Lysates of CRL1807 cells overexpressing adenovirus LacZ control and PAICS were used to check the expression of PAICS, E-cadherin, and vimentin.

For CRCs, loss of E-cadherin is associated with the initiation of the EMT [26]. The transcription factors, SNAI1, SLUG, ZEB1, and ZEB2, that are involved in EMT, repress E-cadherin to promote the mesenchymal features of cancer cells [27]. PAICS knockdown reduced the mRNA transcripts of these markers (Figure 6A). The upregulation of E-cadherin and the downregulation of vimentin are indicators of the suppression of the malignant phenotypes of cells [28,29]. To determine if PAICS knockdown had an effect on the levels of E-cadherin and vimentin, these proteins were evaluated by immunoblot analysis. Upon PAICS knockdown, higher levels of E-cadherin and lower levels of vimentin were evident in the three types of CRC cells (Figure 6B). To test whether E-cadherin protein expression was higher in HCT116^p53-wt^ cells with PAICS knockdown, immunofluorescence assays were performed with a validated antibody; an increase in E-cadherin levels was evident (Figure 6C). We also performed immunoblot analysis on extracts of tumors excised from mice to which cells with PAICS knockdown had been administered and found an increase in E-cadherin expression as compared to controls (tumors from mice treated with NT shRNA) (Figure 6D), while vimentin levels were decreased (Figure S5E). Furthermore, upon transient PAICS overexpression in SV-40 transformed CRL1807 colon cells, the levels of E-cadherin were less and those of vimentin were higher, indicating the involvement of PAICS in the regulation of these levels (Figure 6E). These observations pointed to a function of PAICS in modulating the EMT in CRC cells.

### 2.8. In CRCs, miR-128 Targets and Regulates PAICS

**Figure 7:**
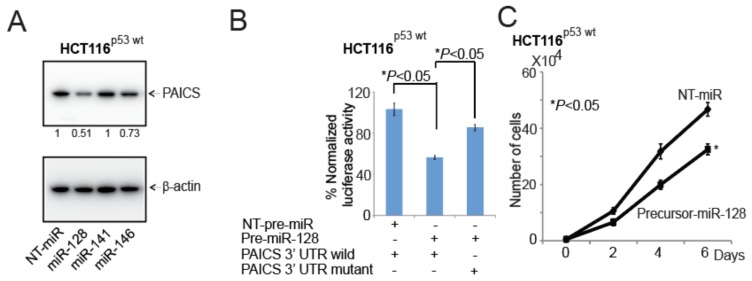

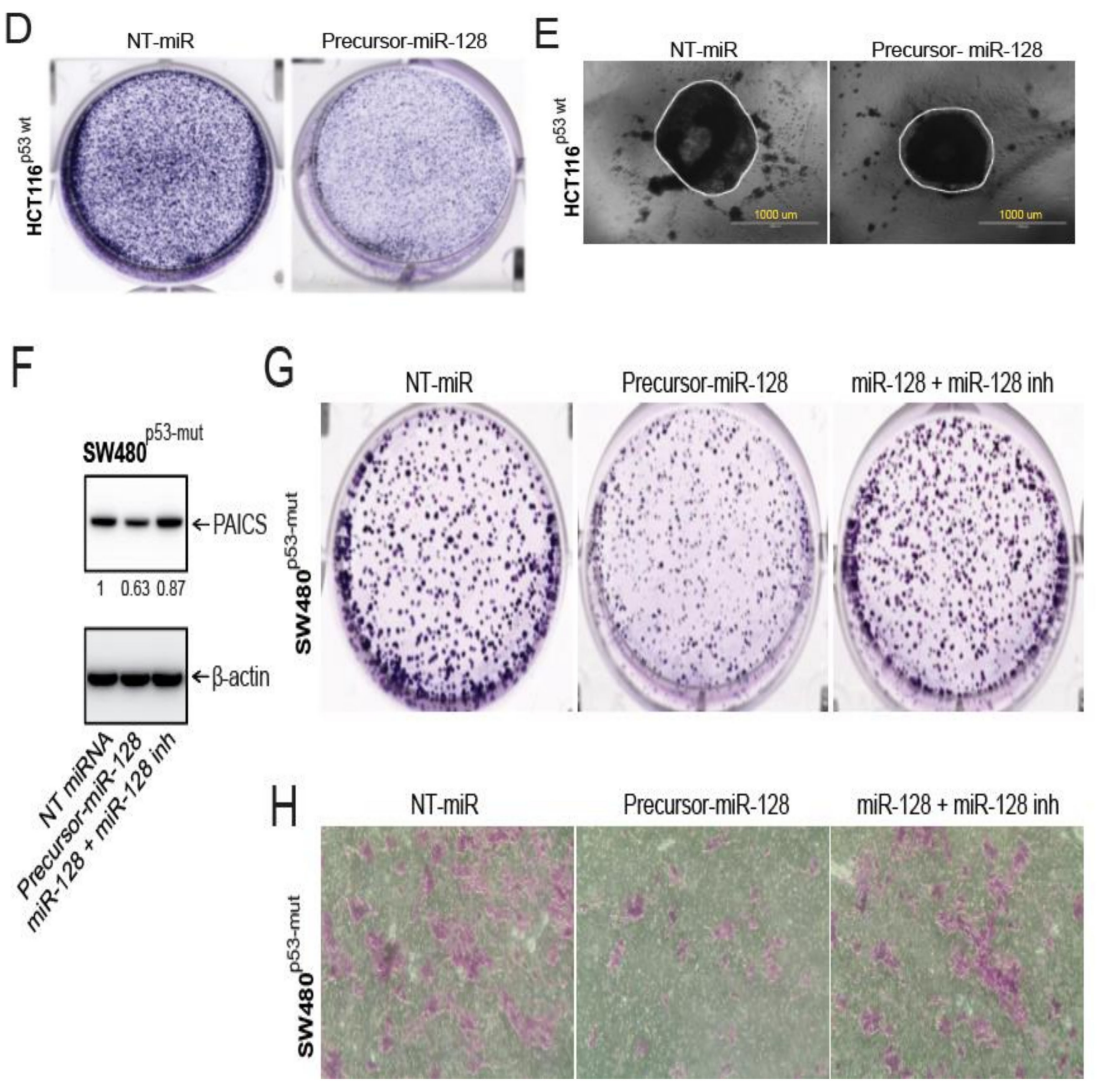
PAICS in CRC is regulated and targeted by miR-128. (**A**) HCT116^p53-wt^ was transfected with NT-pre-miR, miR-128, miR-141, or miR-146 followed by the immunoblot analysis of PAICS protein expression. The numerical values below the blots indicate the normalized ratio of the PAICS/β-Actin. (**B**) The precursor miR-128 or NT pre-miR was co-transfected with luciferase constructs of either PAICS-3’UTR wild-type or mutant. The transfection of HCT116^p53-wt^ cells was accomplished with either NT-pre-miR or miR-128 to assess (**C**) cell proliferation, (**D**) colony formation, and (**E**) spheroid formation; scale bar—1000 µm. (**F**) An immunoblot analysis to show the restoration of the PAICS levels after the ectopic expression of miR-128 followed by co-transfection of precursor-miR-128 and inhibitor of miR-128 in SW480^p53-mut^ cells. Transfections were performed to assess the (**G**) colony formation and (**H**) cell invasion capacity.

As predicted by informatics analyses (TargetScanHuman), miR-128, miR-141, or miR-146 are potential targets of PAICS. Thus, we performed transient transfections with precursor-miR-128, miR-141, or miR-146 in HCT116p53-wt cells to validate the effects of these miRNAs in the regulation of PAICS expression using Western Blot analysis. The results show that PAICS expression was lower after precursor-miR-128 overexpression as compared to NT-miR or other microRNAs (Figure 7A). Moreover, in bladder cancer, it was demonstrated, as we observed, that miR-128 was one of the regulating miRNAs of PAICS [16]. In various cancer types, miR-128 functions as a tumor suppressor, targeting epidermal growth factor receptor in lung cancer [30], polycomb complex protein BMI1 in prostate cancer [31], PAICS in bladder cancer [16], and E2F transcription family member E2F3a in glioblastoma [32]. To determine whether miR-128 binds to the 3’-UTR of PAICS, HCT116^p53-wt^ cells were transfected with miR-128 and pMir-REPORT-PAICS 3′-UTR plasmids. For cells transfected with miR-128, there was a decrease in luciferase activity relative to the NT miR; luciferase activity was unchanged when the miR-128 target sites were mutated (Figure 7B). Furthermore, upon the addition of precursor-miR-128 to this cell line, there was less cell proliferation (Figure 7C), colony formation (Figure 7D), and spheroid formation (Figure 7E; Figure S6A). We further validated this observation and performed a rescue experiment in SW480^p53-mut^ cells. We transfected cells with precursor-miR-128 and rescued this ectopic overexpression with miR-128 inhibitor. The ectopic expression of precursor-miR-128 significantly decreased the expression of PAICS levels in SW480^p53-mut^ cells, while co-transfection of precursor-miR-128 and its inhibitor restored PAICS levels (Figure 7F). The expression of vimentin in these lysates showed lower levels in cells treated with precursor-miR-128; the suppressed levels were rescued by the addition of an miR-128 inhibitor (Figure S6B). Furthermore, miR-128 overexpression reduced the colony formation (Figure 7G) and invasion ability (Figure 7H) while the miR-128 inhibitor reverted the malignant phenotypes of SW480^p53-mut^ cells. These data show that, in CRC cells, miR-128 targets and regulates PAICS expression and acts as tumor suppressor microRNA.

### 2.9. PAICS Expression in CRC is Regulated by Myc

**Figure 8:**
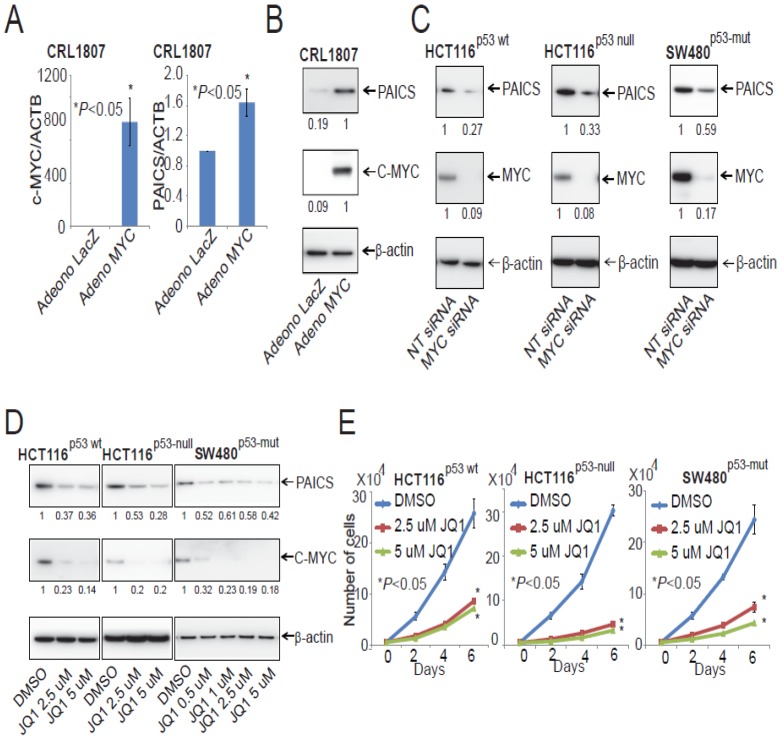

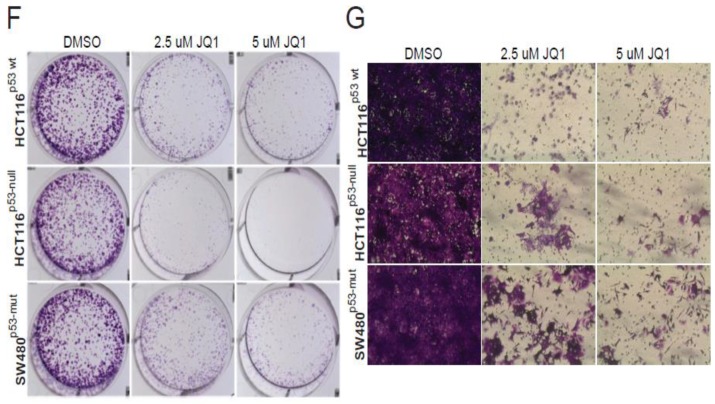
PAICS is Myc-dependent in CRC. Infections with lacZ control and Myc adenovirus in CRL1807 cells were performed followed by (**A**) qPCR analysis and (**B**) immunoblot analysis. (**C**) PAICS and Myc protein levels showed by Immunoblot analysis after Myc knockdown in HCT116^p53-wt^, HCT116^p53-null^, and SW480^p53-mut^ cells. (**D**) Immunoblot analysis of PAICS and C- Myc after JQ1 treatment of HCT116^p53-wt^, HCT116^p53-null^, and SW480^p53-mut^ cells. Cell proliferation (**E**), colony formation (**F**)**,** and invasion (**G**) were assessed after treatment of CRC cells with JQ1.

In prostate cancers, the oncogenic protein Myc is involved in the regulation of PAICS expression [33]. To determine if Myc has a function in PAICS expression in CRC, Myc was overexpressed in SV-40 transformed CRL1807 colon cells by use of an adenovirus. As determined by qPCR and immunoblot analysis, PAICS levels were upregulated after Myc overexpression (Figure 8A,B), demonstrating a function for Myc in regulating PAICS expression. This observation is in line with the results of previous studies, which show that Myc binds to the PAICS promoter [15,17]. Furthermore, we validated the Myc regulation of PAICS by investigating whether the knockdown of Myc via siRNA can affect PAICS expression. Upon siRNA-mediated Myc knockdown, we observed decreased expression of the PAICS protein, suggesting that Myc regulates PAICS expression in CRC cells (Figure 8C). To target the Myc-mediated expression of PAICS, CRC cells (HCT116^p53-wt^, HCT116^p53-null^ and SW480^p53-mut^) were treated with an inhibitor of bromodomain and extra-terminal motif (BET) proteins, JQ1, which reduces Myc expression [15]. The analyses showed lower Myc and PAICS expression after JQ1 treatment (Figure 8D). We further checked for expression of vimentin in JQ1 treated SW480^p53-mut^ cells and found decreased levels (Supplementary Figure S6C). CRC cells treated with JQ1 also showed less cell proliferation (Figure 8E), colony formation (Figure 8F), and invasion (Figure 8G). These data demonstrate that the expression of PAICS is dependent upon Myc expression and is targeted by the BET-bromodomain inhibitor, JQ1.

## 3. Discussions

CRC, a heterogeneous disease involving numerous genomic and epigenetic alterations, is a major cause of cancer-related deaths in the USA [1,34]. The clinical benefits of current chemotherapy and targeted therapies, while useful, are short-lived and restricted to a small subset of patients. Since the development of drug resistance is a prominent clinical problem, efforts are required to develop a range of therapeutic targets and strategies to overcome resistance [35]. New therapeutic approaches and combinatorial treatment therapies are needed to target components of signaling pathways that drive CRC initiation and progression. Advances in tumor DNA and RNA sequencing allow for the evaluation of patient data for the identification of such targets.

Elevated nucleotide pools, formed primarily through the de novo synthesis pathway, are characteristics of many cancer cells and are required for diverse cellular functions [36]. In CRCs, de novo nucleotide synthesis and metabolism regulated by Myc and Rb/E2F transcription factors are associated with uncontrolled proliferation [37]. PPAT, GART, PAICS, ADSL, and ATIC are enzymes involved in the de novo purine biosynthesis pathway. Overexpression of PPAT and ADSL is associated with poor survival of patients with brain tumors [38]. The knockdown of ADSL, GMPS, and PRPS1 decreases brain tumor initiating cell maintenance in both immunodeficient and immunocompetent mouse models [38]. Lomotrexol and AG2034 are GART inhibitors that reduce tumor growth but cause high toxicity [39]. Recently, new GART inhibitors, PY873, PY899, and DIA, have been developed but are yet to be evaluated for side-effects and toxicity [40]. Small molecule inhibitors, LSN3213128 and 326203-A, which target 5-aminoimidazole-4-carboxamide ribonucleotide formyltransferase (AICARFT), a subunit of the ATIC enzyme, are being evaluated for treatment of triple-negative breast cancer [41].

We previously reported that PAICS, an enzyme of de novo purine biosynthesis pathway, is associated with lung cancers [14], prostate adenocarcinomas [15], and bladder cancers [16] and correlated with upregulated cancer phenotypes, such as proliferation and invasion. A recently developed small-molecule inhibitor (MRT00252040), which targets PAICS, is currently being evaluated for the treatment of ovarian, breast, and lung cancers [42]. A recent study demonstrated that the RNA expression of PAICS (Oncomine) was significantly higher in CRCs compared to normal/benign colonic tissues, suggesting that PAICS is a candidate biomarker for CRC [43]. To the best of our knowledge, the present study is the first to investigate its expression, functions, and role in CRC progression. Studies of gene expression profiling show the overexpression of PAICS in CRCs [20,22]. Indeed, the de novo purine nucleotide pathway enzymes, PPAT, GART, PAICS, ADSL, ATIC, and PFAS, are upregulated, as shown from independent CRC gene expression profiling datasets analyzed through Oncomine [20,21]. Our analysis shows that the levels of PAICS RNA and protein were elevated in most CRC tissues irrespective of tumor stage or patient race, gender, or age. Furthermore, in our study, increased PAICS expression was found to be significantly associated with poorer 5-year survival of CRC. However, in multivariate analyses, this association was marginally significant. This may be due to the small sample size and low number of events and suggests that larger independent validation studies may aid in determining the survival effect of PAICS over-expression after adjusting for stage, age and other confounders. These observations prompted us to investigate the function of PAICS in CRC growth using cell cultures and animal models. 

Multiple alterations contribute to development of CRCs, but CRC cell lines that demonstrate wt, mutated, or null status for key molecules (e.g., KRAS, BRAF, PTEN) are not available. However, well-characterized CRC cells for p53 (wt, null, and mut) and microsatellite instability are available. Moreover, most CRCs exhibit p53 mutations (more than 50%) and microsatellite stability (MSS) (about 85% of sporadic CRCs). Furthermore, MSS-CRCs have a proficient mismatch repair (MMR) mechanism that contributes to aggressive behavior of CRCs. Since the function of PAICS in the background of p53 and microsatellite status has not been studied for any other malignancy, a demonstration of a functional connection between these two molecular determinants with PAICS is relevant for CRCs. The silencing of PAICS in CRC cells resulted in reduced cancer cell proliferation, colony-forming, invasion, and spheroid-forming capacity. These observations were made for cells with or without tumor suppressor p53 mutations, suggesting that PAICS-regulated cellular functions are not associated with p53 status. Moreover, growth inhibition after PAICS knockdown was not dependent on the microsatellite status of CRC cells. PAICS knockdown reduced CRC growth, suggesting a role for this gene function. Furthermore, our studies with mice reported the role of PAICS in metastasis for the first time and showed less CRC metastasis upon PAICS knockdown in cancer cells. In particular, PAICS depletion reduced CRC cell dissemination to bone and liver.

In CRCs, PAICS is involved in regulating the EMT, which is involved in regulation of cancer cell invasion and metastasis [44]. In CRCs, the loss of E-cadherin and gain of vimentin are implicated in cancer progression, and metastasis and are associated with a poor prognosis [45]. A previous study showed that the histone methyltransferase, EZH2, mediates transcriptional silencing of E-cadherin by trimethylation of H3 lysine 27 [46]. In the present study, PAICS knockdown induced E-cadherin expression and reduced vimentin expression, indicating the loss of invasive mesenchymal characteristics and the gain of epithelial phenotypes. However, recent studies have challenged the requirement of the EMT for metastasis. Targeting EMT inducers, such as Paired-related homeobox protein 1 (Prrx1), allows cancer cells to acquire properties of metastatic colonization [47,48] and suggest that, for metastasis, the role of the EMT is context-dependent [49]. Moreover, a complex network of transcriptional regulators and molecular signals control the EMT process [50]. In the current study, the high expression of PAICS supports the concept that the EMT contributes to metastasis of CRCs. However, the transformation of cancer cells from the epithelial to mesenchymal and mesenchymal to epithelial (MET) states is required for metastasis [51]. Thus, future studies are needed to demonstrate the relevance of the MET in the metastatic colonization of CRCs that overexpress PAICS.

The broad metabolic reprogramming and signaling associated with cancers has recently led to new approaches for targeting cancer metabolism. Transcription factors such as TP53, Myc, and others mediate oncogenic signaling pathways and regulate metabolic reprogramming in neoplastic [52] and invasive lesions of the colorectum [53]. Furthermore, these signaling pathways control the expression of genes necessary for cancer cell proliferation and energy metabolism. Therapeutic approaches, such as inhibitors of PI3K signaling, are being tested in pre-clinical and clinical studies [54]. Our previous study shows that, in prostate cancers, the bromodomain inhibitor JQ1 decreases PAICS expression by interfering with Myc binding at its promoter [15]. The current observation that ectopic overexpression of Myc in SV-40 transformed colon cells (CRL1807) increased PAICS expression while MYC knockdown in CRC cells decreased PAICS expression suggesting a role for Myc in regulating its expression. Our results indicate that, in CRCs, PAICS was regulated by Myc and decreased EMT by JQ1 treatment through downregulation of PAICS. Thus, the targeting of PAICS with JQ1 could be of therapeutic value. In addition, we also observed a role for miR-128 in the regulation of PAICS expression in CRC cells and showed that miR-128 targets and regulates the expression of PAICS, leading to decreased CRC malignant phenotypes in a rescue experiment. The tumor suppressor role of miR-128 was also reported in bladder cancer [16], lung cancer [30], prostate cancer [31] and glioblastoma [32]. Moreover, low levels of miR-128 are associated with CRC progression and predict a poor overall survival of CRC patients [55].

## 4. Materials and Methods

### 4.1. Colorectal Tissue Specimens

The formalin-fixed, paraffin-embedded (FFPE) archival tissue blocks and frozen specimens were collected from the Anatomic Pathology Division of the University of Alabama at Birmingham (UAB). All samples were collected and utilized with the prior approval of the UAB Institutional Review Board (IRB number: 020830005). The FFPE tissue blocks with invasive CRCs and their corresponding normal/benign tissues were procured for 116 CRC patients (Stage I, 17; Stage II, 36; Stage III, 45; Stage IV, 18) who had undergone surgical resection at UAB Hospital. Additionally, tissue extracts from normal and CRC tissues were prepared from frozen specimens for immunoblot analyses. RNA samples were isolated from FFPE tissues that were macro-dissected from tumors and their corresponding normal tissues as well as frozen CRC samples and were analyzed for PAICS expression by quantitative real-time PCR (qRT-PCR) (Tables S1–S3). Tissue sections from FFPE archival tissue blocks were used for immunohistochemical (IHC) analyses.

### 4.2. Cell Lines and Transfections

CRC cell lines HCT116^p53-wt^, HCT116^p53-null^, SW480^p53-mut^, and HT29^p53-mut^ were grown in McCoy’s medium (Corning™ Cellgro™, Fisher Scientific Co., Pittsburgh, PA, USA); SV40 transformed CRL1807 colonic epithelial cells were grown in DMEM high-glucose medium supplemented with 10% fetal bovine serum (FBS, Invitrogen, ThermoFisher Scientific, Carlsbad, CA, USA) and penicillin-streptomycin in a 5% CO_2_ incubator. The cells were regularly screened for mycoplasma, and cell line identification was accomplished by analysis of a pattern of defined short tandem repeats. pGreenPuro^TM^ shRNA expression lentivectors (Systembio, Palo Alto, CA, USA) were used to package lentiviruses against the *PAICS* gene by the UAB Neuroscience NINDS Protein Core (P30 NS47466) and stable cell lines were developed using puromycin selection as lentivector have puromycin selection resistance marker. PAICS shRNA sequences are listed in Supplementary Table S4. We used precursor-miR-128 (Assay ID: PM11746; ThermoFisher Scientific, Carlsbad, CA, USA) for the transient transfection of CRC cells and rescued the effects of miR-128 with a miR-128 inhibitor (Assay ID: MH11746; ThermoFisher Scientific, Carlsbad, CA, USA). For Myc knockdown, Dharmacon’s On target plus smartpool siRNA against Myc (Cat no. L-00328202-0005; Horizon Discovery; Lafayette, CO, USA) was used to transfect CRC cells using Lipofectamine 3000 (ThermoFisher Scientific, Carlsbad, CA, USA).

### 4.3. Gene Expression from TCGA

Oncomine (http://www.oncomine.org) [20] and UALCAN (http://ualcan.path.uab.edu) [22] were used to obtain the TCGA gene expression profile of PAICS in CRCs and normal colon/rectum samples. The statistical significance of the results (*p*-values) was provided by these websites; *p*-values < 0.05 were considered statistically significant. The correlation between the mRNA levels of PAICS and pathologic stage, patient race/ethnicity, age, and gender was analyzed for CRC patients using UALCAN.

### 4.4. Quantitative Real-Time PCR (qPCR)

RNeasy mini kits (Zymo Research, Irvine, CA, USA) were used to isolate total RNA from CRC tissues and cell lines. Superscript III Reverse Transcriptase (Invitrogen, Carlsbad, CA, USA) was utilized to convert RNA into complementary DNA. SYBR green qPCR was performed with primers synthesized by Integrated DNA Technologies (Coralville, IA) to determine the mRNA expression of genes as described [56]. ACTB was used as a normalizing control, and the data were analyzed using the 2^(-ΔΔCt) method. A list of primers is in Table S5.

### 4.5. Immunohistochemical (IHC) Analysis 

IHC analyses were performed for CRC tissue sections as described earlier [57]. To evaluate PAICS expression, IHC was used with a mouse monoclonal antibody against human PAICS (Cat# GTX83950, GeneTex, Irvine, CA, USA) and further probing with ImmPRESS horseradish peroxidase anti-mouse IgG (Cat# MP-7402, Vector laboratories, Burlingame, CA, USA) as a secondary antibody. Immunoreactivity was assessed with diaminobenzidine (Cat#SK-4100, Vector laboratories, Burlingame, CA, USA). Vector Hematoxylin QS (Cat#H-3404, Vector laboratories, Burlingame, CA, USA) was used as the counterstain.

### 4.6. Immunoblot Analysis

NuPAGE™ 4–12% Bis-Tris Midi Protein Gels, 20-well (Invitrogen, ThermoFisher Scientific, Carlsbad, CA, USA) were used to separate proteins, which were transferred onto Immobilon-P PVDF membranes (EMD Millipore, Billerica, MA, USA), followed by incubation for 1 hour in blocking buffer (Tris-buffered saline, 0.1% Tween [TBS-T], 5% nonfat dry milk) as described previously [58]. The membranes were incubated overnight at 4 °C with a primary antibody. After washing with TBS-T, the blots were incubated with horseradish peroxidase-conjugated secondary antibody, and the signals were visualized with Luminata^TM^ Crescendo chemiluminescence Western Blotting substrate according to the manufacturer’s protocol (EMD Millipore, Billerica, MA, USA). A list of primary antibodies is in Table S6. The quantitative densitometric analysis was performed using Image J.

### 4.7. RNA Interference

CRC cells were transfected with pGreenPuro^TM^ shRNA expression lentivectors (Systembio, Palo Alto, CA, USA) consisting of PAICS shRNA or non-targeting (NT) controls, and stable cell lines were generated by selection with medium containing 1 μg/mL puromycin (Life Technologies, ThermoFisher Scientific, Carlsbad, CA, USA) as described previously [16]. Stable cells were used for RNA isolation, immunoblot analyses, and cell-based assays.

### 4.8. Cell Proliferation Assay

To assess cellular proliferation, 5 × 10^3^ CRC cells with stable PAICS knockdown or transfected with a scramble control were seeded in triplicate wells of 24-well plates as described previously [15]. After trypsinization, cell numbers were counted with a Z2 Coulter particle counter (Beckman Coulter, Brea, CA, USA) at 2, 4, and 6 days.

### 4.9. Clonogenic Assay

For assay of colony formation, 1 × 10^3^ CRC cells with stable PAICS knockdown or transfected with a scramble control were seeded into 6-well plates in triplicate wells as described [59]. At 10 days after seeding, the cells were fixed with 5% glutaraldehyde in phosphate-buffered saline (PBS) and stained with crystal violet (Sigma-Aldrich, St. Louis, MO, USA). After washing cells with PBS, photographs of the colonies were made with an Amersham Imager 600RGB (GE Healthcare Life Sciences, Pittsburgh, PA, USA).

### 4.10. Invasion Assay 

Corning BioCoat Matrigel invasion chambers (Cat#354480, Corning, NY, USA) were used to evaluate cell invasion and thereby to determine the involvement of PAICS in the malignant properties of CRC cells, as described previously [60]. In 500 μl of serum-free medium, CRC cells (5 × 10^4^) with stable PAICS knockdown or transfected with a scramble control were layered on the 8-μm pore inserts of the Transwell membranes in triplicate. In the lower chambers, medium (750 μL) supplemented with 10% FBS was used as a chemoattractant. After 48 hours, the non-invading cells and the Matrigel matrix were removed with a cotton swab. The cells that invaded through the Matrigel matrix and migrated to the lower side of the membrane were fixed with 5% glutaraldehyde in PBS and were then stained with crystal violet followed by imaging with a phase-contrast microscope. 

### 4.11. Wound Healing Assay

Cell motility was measured by a wound-healing assay as described [61]. CRC cells (1 × 10^6^) with stable PAICS knockdown and cells transfected with a scramble control were seeded on 35-mm Petri dishes in triplicate. On the monolayers of confluent cells, artificial wounds were created with the tip of a 200-μL pipet. At 0 and 24 hours, the dishes were washed with PBS, and photomicrographs were taken with an inverted phase-contrast microscope and a 4× objective. 

### 4.12. 3D Spheroid Model

Spheroids of CRC cells with PAICS knockdown were established by use of the Cultrex® 3D spheroid BME cell invasion assay (Cat# 3500-096-K, Trevigen, Gaithersburg, MD, USA) as described previously [16]. As per the manufacturer’s instructions, 5 × 10^3^ cells (45 µL) were seeded in 96-well plates in triplicate with 5 µL of Spheroid Formation ECM followed by centrifugation at 200× *g* for 3 minutes and incubated in a 37 °C, 5% CO_2_ incubator to promote spheroid formation. After 72 hours of incubation, 50 µL of invasion matrix was added to the wells, and, to position spheroids within the invasion matrix toward the middle of the wells, centrifugation was accomplished at 200× *g* for 3 minutes at 4 °C. After 1 hour, warm medium (100 µL) supplemented with FBS was added. The plates were incubated for 4 days, and images were taken with a 4× objective.

### 4.13. Immunofluorescence Staining

CRC cells (2 × 10^3^) were grown overnight on a Lab-Tek® II CC2 Chamber slideTM System (Cat#154917, Nunc, Rochester, NY, USA) as described previously [16,62]. Cells were fixed with chilled methanol for 10 minutes and washed with PBS. The samples were blocked with goat serum for 30 min and then incubated with primary antibodies for 1 h, followed by incubation with secondary antibodies for 1 h. Samples were mounted with Prolong® Gold Antifade reagent with 4’,6-diamidino-2-phenylindole (DAPI, Cat#P36931, Life technologies, Eugene, OR, USA). Confocal images were acquired by the UAB High-Resolution Imaging Facility using a 60× lens Nikon A1, High Speed Laser Confocal Spectral Imaging microscope (Nikon Instruments Inc., Melville, NY, USA). The detector gains were constant for all acquisitions.

### 4.14. Chick Chorioallantoic Membrane (CAM) Assay

A CAM assay was performed to assess growth of tumor cells in fertilized chicken eggs (Charles River Laboratories, North Franklin, CT), as described previously [15,59]. Briefly, eggs were incubated for 10 days in a rotary incubator at 37 °C and 58–60% humidity. CRC cells (1 × 10^6^), stably exhibiting NT shRNA or stably inhibiting PAICS by shRNA1, were applied to the CAM in 50 µL of culture medium. On the 18th day of embryonic growth, the tumors were harvested and weighed. Of the 10 eggs per group, two from each group were non-viable.

### 4.15. Mouse Model of Colorectal Tumor Growth and Metastasis

The UAB Institutional Animal Care and Use Committee (IACUC) approved the study and confirmed that regulatory standards were followed (Animal Project Number: 21182). For the investigation of the function of PAICS on tumor growth, HCT116^p53-null^ cells (2 × 10^6^ cells in 50 µL of incomplete media, without FBS, and 50 µL of Matrigel) transfected with PAICS shRNA1 or with NT shRNA were injected subcutaneously into the right dorsal flanks of 6-week-old NOD/SCID/IL2γ-receptor null (NSG) mice (*n* = 7 for each group) as described [15,63]. The mice were monitored daily for tumor growth, and tumors were measured three times a week by use of a Vernier caliper. Tumor volumes (mm^3^) were calculated with the formula (0.52) × (length) × (width^2^). At end of the experiment, the tumors were excised from euthanized mice, photographed, and weighed. For the metastasis assay, HT29 cells (0.5 million cells in 100 µL of incomplete media without FBS) stably expressing luciferase with and without PAICS knockdown were injected into the lateral tail-veins of NSG mice. The bioluminescence imaging of animals was performed weekly by injecting luciferin and imaging after 10 minutes using an IVIS Lumina III (Perkin Elmer, Waltham, MA, USA). Metastasized organs were excised from animals injected with luciferin, and ex vivo bioluminescence signals were assessed.

### 4.16. Positron-Emission Tomography (PET) Imaging 

^18^F-Fluorodeoxyglucose (FDG) was used for PET imaging performed as described by Kang et al. [64]. The mice were administered FDG intravenously one hour before whole-body PET, and, with mice under inhalation anesthesia (2% isoflurane), images were captured over 20 min.

### 4.17. Bone Marrow Cultures

Mouse bone marrow cells were cultured as described by Gutierrez-Uzquiza and colleagues [24]. The bone marrow cells were flushed from the femurs and tibias of mice injected with cells transfected with NT shRNA or PAICS shRNA and cultured with puromycin-containing media to select cancer cells that exhibited green fluorescent protein (GFP) and puromycin-resistant genes. GFP and bright field images were taken with an inverted microscope.

### 4.18. Statistical Analysis

For experiments with cell lines, Student’s t-test was used to perform comparisons of mean values. *p*-values of <0.05 were considered statistically significant. Data were expressed as means ± standard deviation for triplicates. Optimal cut-point for PAICS expression was determined using a maximally selected rank statistic approach [65]. A qPCR threshold value of 2.01 of PAICS expression only in tumors was used as a cut-off to binarize tumors into high and low expression groups. The survival analysis was done in a subset of 87 patients with complete follow up and vital status information (Table S2). Participants with less than 1 month follow up were excluded. Kaplan-Meier log-rank tests were performed to determine univariate differences in survival between high and low expression groups. Stage-adjusted survival analysis was conducted with a Cox proportional hazards model. Proportionality assumptions were tested.

## 5. Conclusions

Overall, our results show that the de novo purine metabolic enzyme PAICS is overexpressed in human CRCs, and its expression is regulated by miRNA-128 and Myc. PAICS is required for tumor growth and metastasis, and PAICS knockdown decreases the dissemination of CRC cells to liver and bone. In addition, treating CRC cells with a BET bromodomain inhibitor, JQ1 that targets Myc, reduces PAICS, highlighting the dependency of PAICS expression on Myc. Furthermore, PAICS regulates the EMT-related protein E cadherin. A high expression of PAICS is associated with a poor survival of CRC patients. Thus, PAICS is a potential therapeutic target of CRC.

## Supplementary Materials

The following are available online at https://www.mdpi.com/2072-6694/12/4/772/s1, Figure S1: Expression of de novo purine biosynthetic enzymes in CRCs. Gene expression profiling showing elevated expression of de novo purine biosynthetic enzymes. The data were acquired from Skrzypczak colorectal data sets. The color scale highlights different expression, as blue represents downregulation, white indicates no alteration, and red shows the upregulation of transcripts, related to Figure 1. Figure S2. PAICS expression in CRC. UALCAN data showing PAICS expression in the race (**A**), gender (**B**), age (**C**), and weight (**D**) of patients. (**E**) qRT-PCR showing PAICS expression in various age groups. (**F**) Immunofluorescence analysis to show the PAICS expression in HT29^p53-mut^; scale bar = 20 µm., related to Figure 1 and Figure 2. Figure S3. PAICS knockdown decreased colony formation, invasion, and spheroid-forming capacity of CRC cells. The graph shows the approximate number of malignant phenotypes formed after PAICS knockdown as compared to NT shRNA. (**A**) Colony formation, (**B**) invasion, and (**C**) spheroid-forming capacity of CRC cells., related to Figure 3. Figure S4. PAICS knockdown reduces motility irrespective of p53 status. Representative images taken at 0 h and 24 h showed reductions in wound-healing capacity of colon cancer cells with PAICS knockdown compared to those transfected with NT shRNA., related to Figure 3. Figure S5. PAICS is involved in CRC tumor growth and metastasis. (**A**) Histogram depicting decreased tumor weights in CAM eggs implanted with cells exposed to PAICS shRNA. * *p* < 0.05, statistically significant (related to Figure 3). (**B**) Representative images showing tumor load beneath the lungs and vertebra in mice injected with cells transfected with control NT shRNA as compared those transfected with PAICS shRNA. Arrows show metastatic lesions in mice injected with cells transfected with NT shRNA. (**C**) Metastatic tumor procured from the thoracic neck area and vertebra. (**D**) H&E of thoracic metastatic lesions (Related to Figure 4). (**E**) Tumor lysates obtained from xenografts were analyzed for vimentin; β-actin was used as a loading control (related to Figure 6). Figure S6. miR-128 regulates PAICS expression in CRCs. (**A**) Graph showing spheroid formation after transient transfection of cells with precursor-miR-128. (**B**) Vimentin levels were measured in SW480^p53-mut^ cells treated with precursor-miR-128; rescue experiments were performed by addition of an miR-128 inhibitor. (**C**) Western blot analysis showing vimentin levels in lysates of JQ1-treated SW480^p53-mut^ cells. Table S1: PAICS expression (qPCR) and clinic-pathologic characteristics of CRCs, Table S2: Association between death from CRC, PAICS expression, and clinical variables, Table S3: PAICS expression and 5-year survival: Cox model, Table S4: shRNA sequences used, related to Materials and Methods, Table S5: qPCR Primer sequences used, Related to Materials and Methods, Table S6: List of antibodies used, Related to Materials and Methods.
