# Supplementary material for: PAICS, a Purine Nucleotide Metabolic Enzyme, is Involved in Tumor Growth and the Metastasis of Colorectal Cancer
Source: Cancers (Basel). 2020 Mar 25;12(4):772. doi: 10.3390/cancers12040772 (PMC7226071; doi:10.3390/cancers12040772)

REVISED SUPPLEMENTAL TABLES, FIGURES AND FIGURE LEGENDS

PAICS, a purine nucleotide metabolic enzyme, is involved in tumor growth and metastasis of colorectal cancer

Sumit Agarwal[1], Balabhadrapatruni V. S. K. Chakravarthi[1], Michael Behring[1], Hyung-Gyoon Kim[1], Darshan S. Chandrashekar[1], Nirzari Gupta[2], Prachi Bajpai[1], Amr Elkholy[1], Sai A. H. Balasubramanya[1], Cherlene Hardy[3], Sameer Al Diffalha[1], Sooryanarayana Varambally [1,4,#], and Upender Manne [1,4,#,*]

[1]Department of Pathology, University of Alabama at Birmingham,
[2]Department of Chemistry, University of Alabama at Birmingham,
[3]Department of Radiation Oncology, University of Alabama at Birmingham,
[4]O'Neal Comprehensive Cancer Center, University of Alabama at Birmingham, Birmingham, AL Birmingham, AL, 35233, USA
#Share Senior Authorship

*Correspondence to: Upender Manne, Ph.D., Anatomic Pathology Division, Department of Pathology, Wallace Tumor Institute, Room # 420A, University of Alabama at Birmingham, Birmingham, AL 35233, USA; Phone: (205) 996-1654; Email: upendermanne@uabmc.edu

and

Sooryanarayana Varambally, Ph.D., Molecular and Cellular Pathology, Department of Pathology, Wallace Tumor Institute, Room # 430, University of Alabama at Birmingham, Birmingham, AL 35233, USA. Phone: (205) 996-1654, Email: svarambally@uabmc.edu

Running Title: PAICS targeting in colorectal cancer growth and metastasis

Disclosure of Potential Conflicts of Interest: No potential conflicts of interest were disclosed.

Supplementary tables, Related to Methods

Table S1. Phosphoribosylaminoimidazole carboxylase, phosphoribosylaminoimidazole succinocarboxamide synthetase (PAICS) expression (qPCR) and clinic-pathologic characteristics of colorectal cancers (CRCs).

Pathologic and clinical features	qPCR (%)
All CRCs	82/116 (70.6)
Tumor stages	
Stage I	10/17 (58.8)
Stage II	28/36 (77.7)
Stage III	33/45 (73.3)
Stage IV	11/18 (61.1)
Patient race	
Caucasian	50/70 (74.2)
African American	27/39 (69.2)
Other	5/7 (51.4)
Patient gender	
Male	44/63 (69.8)
Female	38/53 (71.6)
Age groups	
21-40 years	5/7 (71.4)
41-60 years	26/36 (72)
61-80 years	42/57 (73.6)
81-100 years	9/16 (56.2)

Table S2. Association between death from CRC, PAICS expression, and clinical variables

	Alive	Dead	p-value
	(N=77)	(N=10)	
PAICS expression			0.15
- low	46 (59.7%)	3 (30.0%)	
- high	31 (40.3%)	7 (70.0%)	
Age	62.4 ± 14.5	63.6 ± 10.7	0.80
Stage			0.35
- I	13 (16.9%)	0 (0.0%)	
- II	25 (32.5%)	2 (20.0%)	
- III	28 (36.4%)	6 (60.0%)	
- IV	11 (14.3%)	2 (20.0%)	
Sex			0.83
- Female	38 (49.4%)	4 (40.0%)	
- Male	39 (50.6%)	6 (60.0%)	
Race			1.00
- African American	30 (39.0%)	4 (40.0%)	

- Caucasian	47 (61.0%)	6 (60.0%)

Table S3. PAICS expression and 5-year survival: Cox model

	Hazard Ratio	95% CI	P-value
PAICS: high vs. low	4.70	(0.95-23.39)	0.058
Stage: III&IV vs. I&II	2.87	(0.58-14.27)	0.198

Likelihood ratio test= 6.3 on 2 df, p=0.04

Table S4. shRNA sequences used, related to Materials and Methods.

Gene name	Catalog No	Supplier	Sequence
PAICS	shRNA 1<br>shRNA 2	System Biosciences, Mountain View, CA	GUACACUGGUUGAUAUGAA<br>GAAGGGCUCCAAAUGGUAA

Table S5. qPCR Primer sequences used, Related to Materials and Methods.

Gene name	Forward primer	Reverse primer
PAICS	GTGGCAGGCAGAAGTAATGG	CACATCCTGAACTCCCCAGT
SNAI1	TGCCCTCAAGATGCACATCCGA	GGGACAGGAGAAGGGCTTCTC
SLUG	ATCTGCGGCAAGGCGTTTTCCA	GAGCCCTCAGATTTGACCTGTC
ZEB1	GGAAAGCGCTTCTCACACTC	GTCACGTTCTTCCGCTTCTC
ZEB2	TACGGATCCCGAAACGATAC	GGTTTCCATTTTCCCATCCT
c-MYC	CCTGGTGCTCCATGAGGAGAC	CAGACTCTGACCTTTTGCCAGG
ACTB	GCACAGAGCCTCGCCTT	GTTGTCGACGACGAGCG

Table S6. List of antibodies used, Related to Materials and Methods.

Antibody	Application	Dilution	Supplier	Cat. No.
PAICS	IB, IHC	IB, 1:5000<br>IHC, 1:10000<br>IF, 1:500	Genetex, Irvine, CA	GTX83950
E-Cadherin	IB	IB, 1: 2000<br>IF, 1:200	BD Pharmingen, San Diego, CA	610181
Vimentin	IB	IB, 1:1000	Santa Cruz Biotechnology, Santa Cruz, CA	sc-6260
β–Actin-HRP	IB	IB, 1:100000	PTG Labs, Chicago, IL	HRP-60008
CDX2	IF	IF, ready to use	Abcam, Cambridge, MA	ab86949
Arginase	IF	IF, 1:100	Abcam, Cambridge, MA	ab91279
Cytokeratin8+ 18	IF	IF, 1:100	Abcam, Cambridge, MA	ab17139
Alkaline phosphatase	IF	IF, 1:100	Abcam, Cambridge, MA	ab108337
Anti-Mouse IgG HRP	IB	IB, 1:5000	PTG Labs, Chicago, IL	SA00001-1
Anti-Mouse IgG Alexa Fluor 555	IF	IF, 1:1000	ThermoFisher Scientific, Eugene, OA	A21425
Anti-Rabbit IgG Alexa Fluor 555	IF	IF, 1:1000	ThermoFisher Scientific, Eugene, OA	A21430
Anti-mouse IgG Alexa Fluor 647	IF	IF, 1:1000	ThermoFisher Scientific, Eugene, OA	A32728

IB: immunoblotting, IHC: immunohistochemistry, IF: immunofluorescence.

SUPPLEMENTARY FIGURE LEGENDS

Supplementary Figure S1. Expression of *de novo* purine biosynthetic enzymes in colorectal cancers (CRCs). Gene expression profiling showing elevated expression of *de novo* purine biosynthetic enzymes. The data were acquired from Skrzypczak colorectal data sets. The color scale highlights different expression, as blue represents down-regulation, white indicates no alteration, and red shows upregulation of transcripts. Related to Figure 1.

Supplementary Figure S2. PAICS expression in CRC. UALCAN data showing PAICS expression in race (A), gender (B), age (C), and weight (D) of patients. (E) qRT-PCR showing PAICS expression in various age groups. (F) Immunofluorescence analysis to show the PAICS expression in HT29$^{p53\text{-}mut}$; scale bar= 20 µm. Related to Figure 1 and 2.

Supplementary Figure S3. PAICS knockdown decreased colony formation, invasion, and spheroid-forming capacity of CRC cells. The graph shows the approximate number of malignant phenotypes formed after PAICS knockdown as compared to NT shRNA. (A) Colony formation, (B) invasion, and (C) spheroid-forming capacity of CRC cells. Related to Figure 3.

Supplementary Figure S4. PAICS knockdown reduces motility irrespective of p53 status. Representative images taken at 0 hr and 24 hr showed reductions in wound-healing capacity of colon cancer cells with PAICS knockdown compared to those transfected with NT shRNA. Related to Figure 3.

Supplementary Figure S5. PAICS is involved in CRC tumor growth and metastasis. (A) Histogram depicting decreased tumor weights in CAM eggs implanted with cells exposed to PAICS shRNA. *P<0.05, statistically significant (Related to Figure 3). (B) Representative images showing tumor load beneath the lungs and vertebra in mice injected with cells transfected with control NT shRNA as compared those transfected with PAICS shRNA. Arrows show metastatic lesions in mice injected with cells transfected with NT shRNA. (C) Metastatic tumor procured from the thoracic neck area and vertebra. (D) H&E of thoracic metastatic lesions (Related to Figure 4). (E) Tumor lysates obtained from xenografts were analyzed for vimentin; β-actin was used as a loading control (Related to Figure 6).

Supplementary Figure S6. miR-128 regulates PAICS expression in CRCs. (A) Graph showing spheroid formation after transient transfection of cells with precursor-miR-128. (B) Vimentin levels were measured in $SW480^{p53\text{-}mut}$ cells treated with precursor-miR-128; rescue experiments were performed by addition of an miR-128 inhibitor. (C) Western blot analysis showing vimentin levels in lysates of JQ1-treated $SW480^{p53\text{-}mut}$ cells.

Supplementary Figure S1

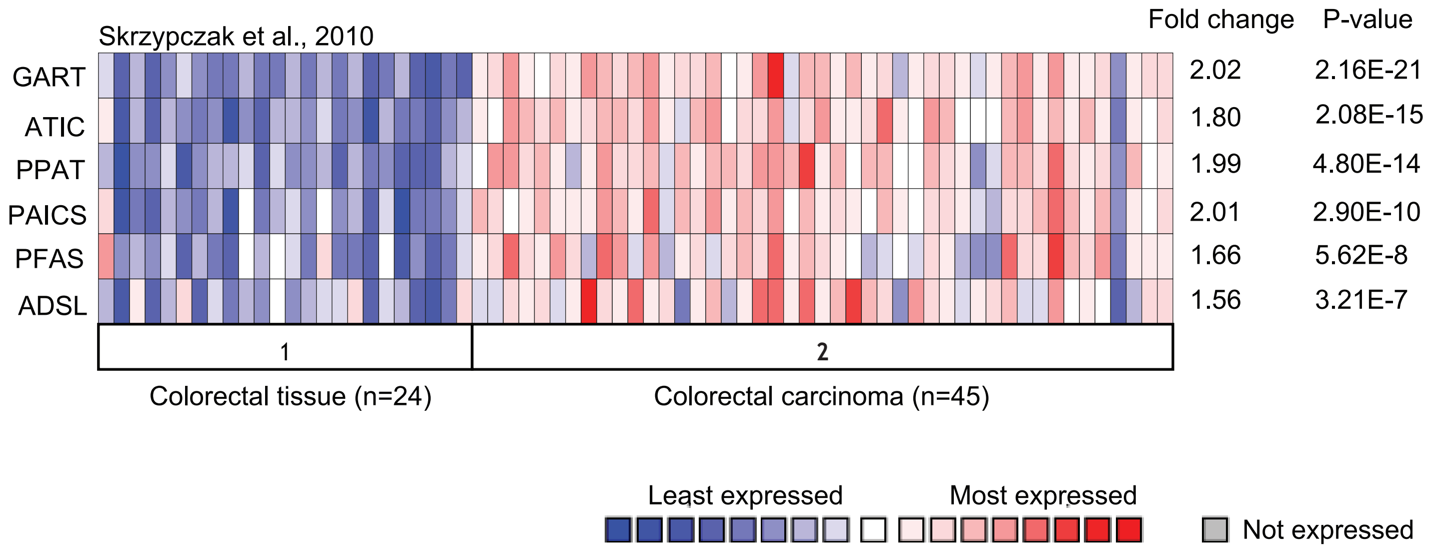

Supplementary Figure S2

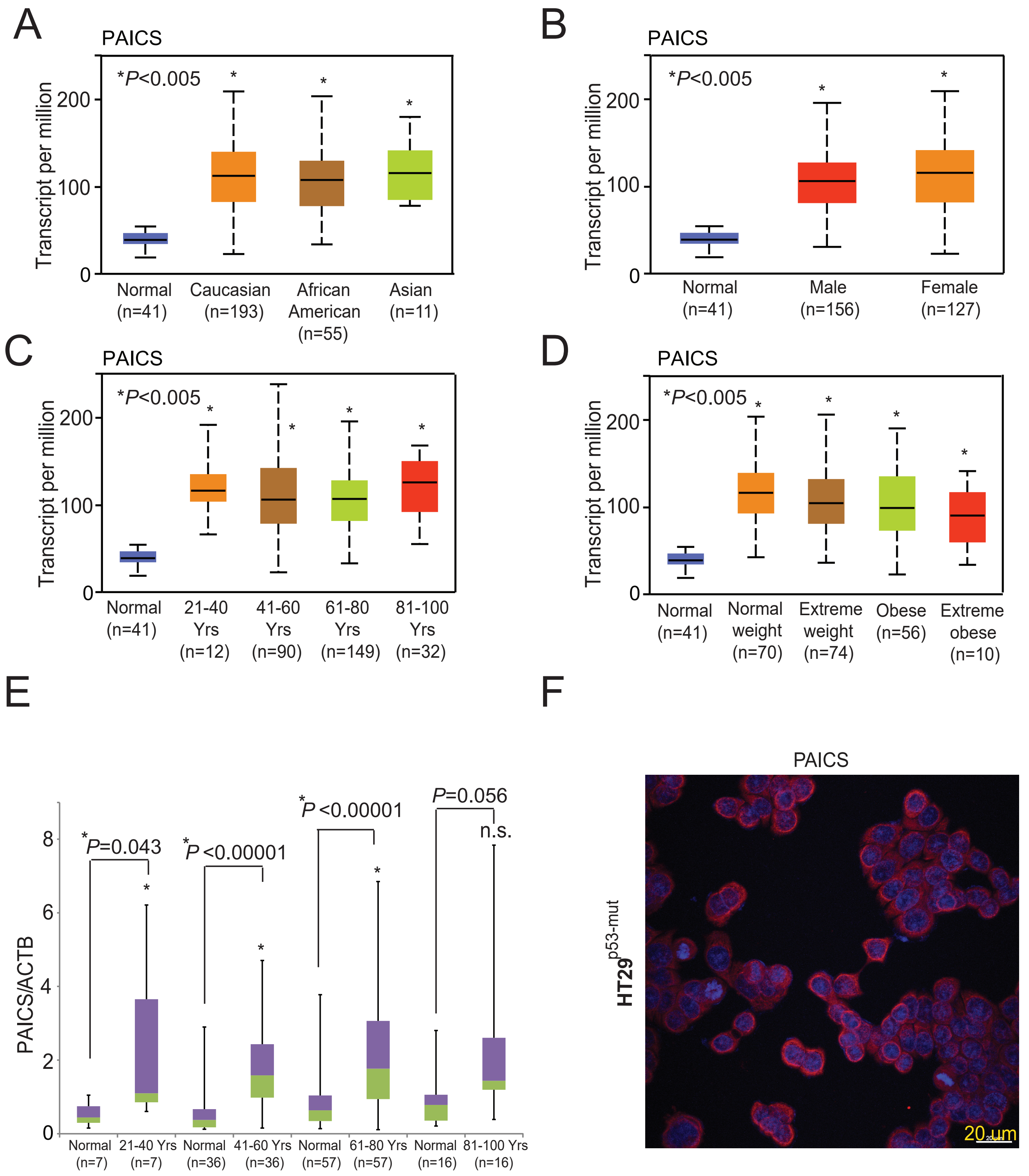

Supplementary Figure S3

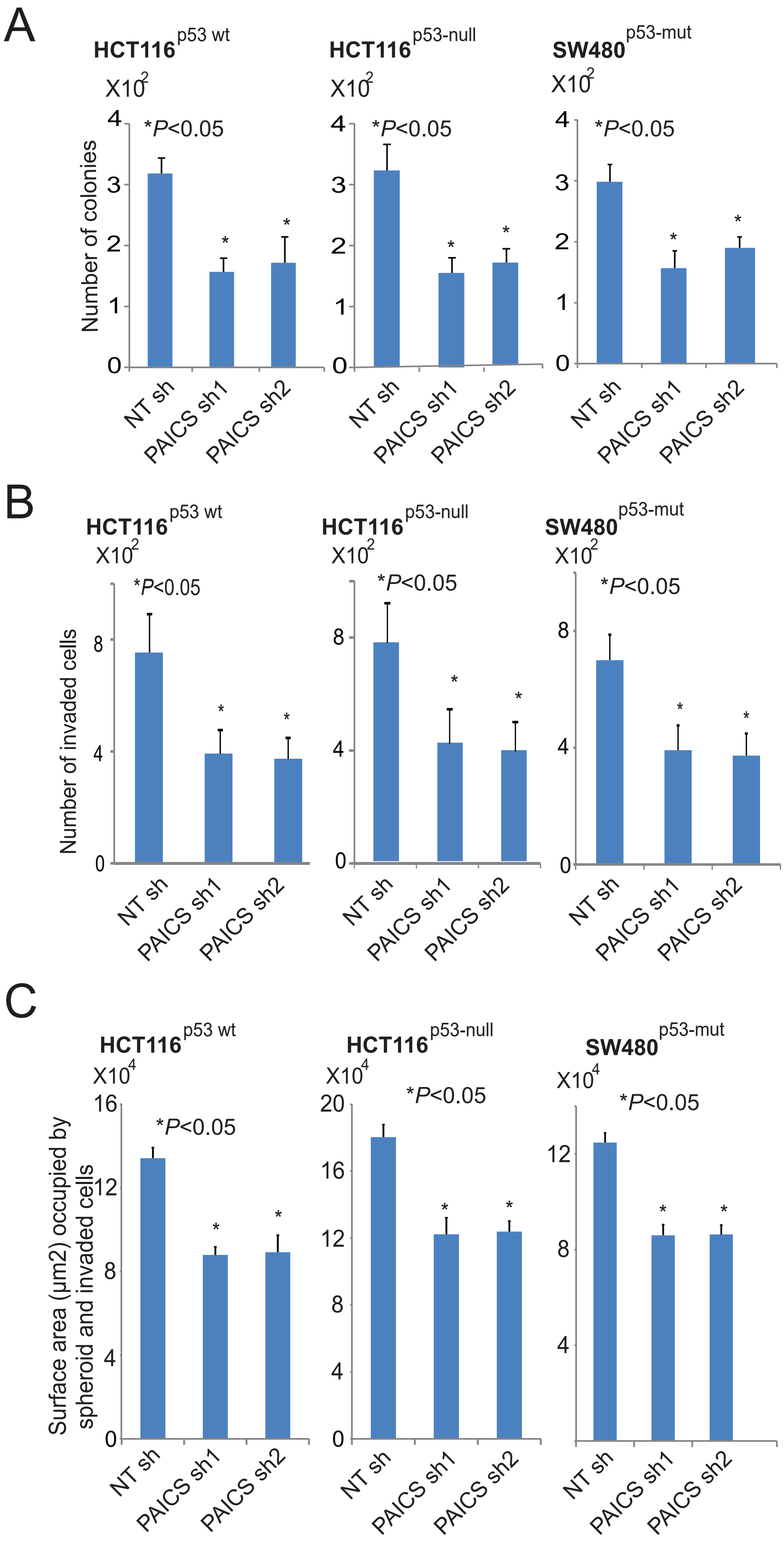

Supplementary Figure S4

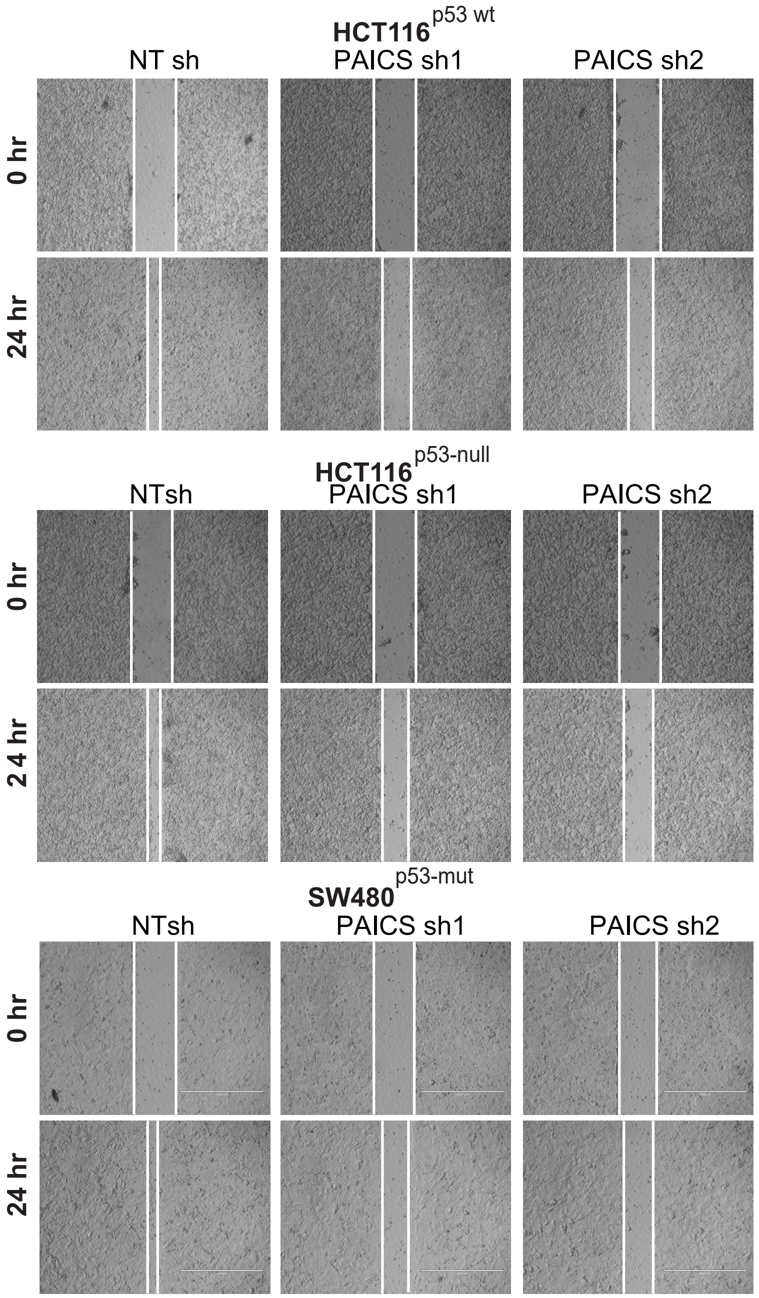

Supplementary Figure S5

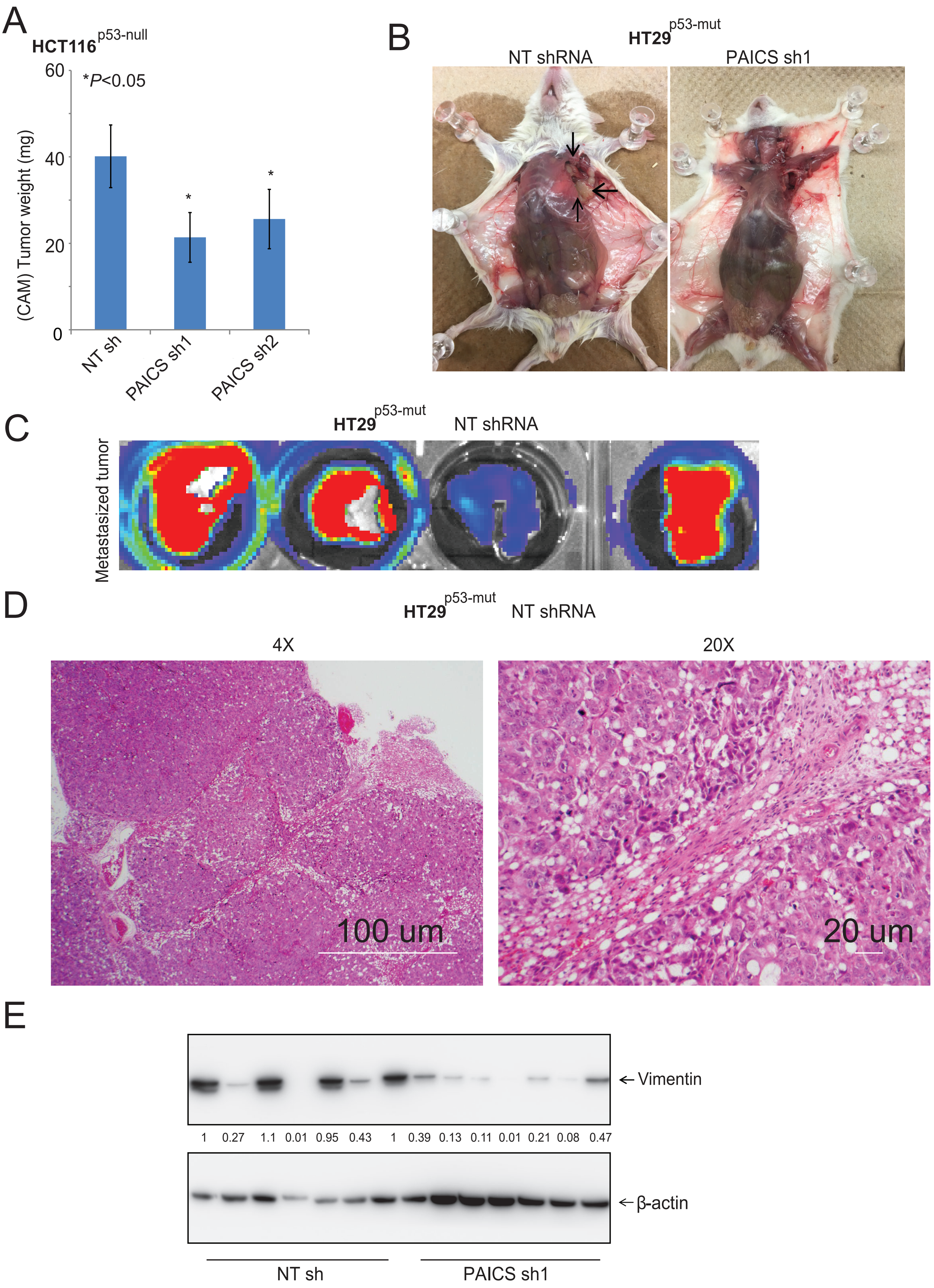

Supplementary Figure S6

A

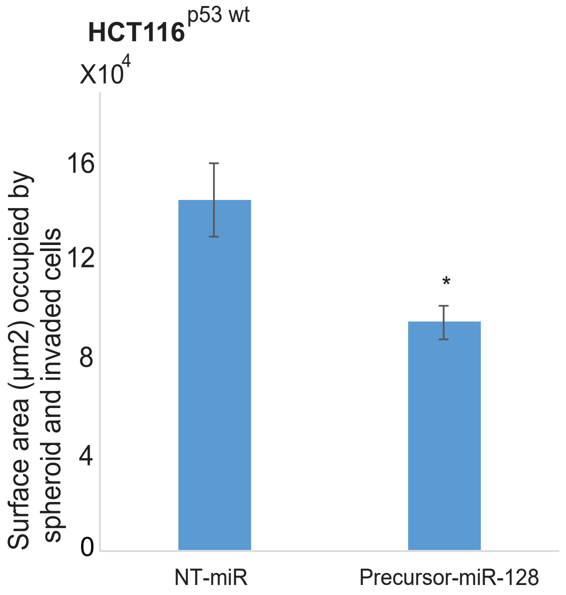

B

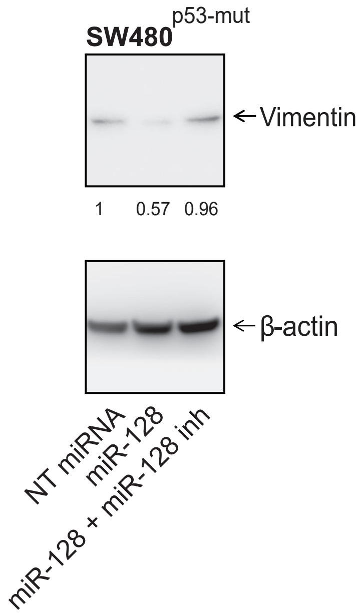

C

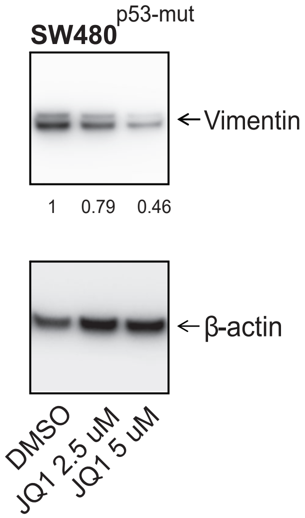

PAICS overexpression in colon cancer tissues, Fig 2A

STAGE I & II

1. 15N STAGE I
2. 15T STAGE I
3. 8N STAGE II
4. 8T STAGE II
5. 23N STAGE II
6. 23T STAGE II
7. 36N STAGE II
8. 36T STAGE II
9. 58N STAGE II
10. 58T STAGE II
11. 68N STAGE II
12. 68T STAGE II
13. 280N STAGE II
14. 280T STAGE II

Raw blots; Fig 2A

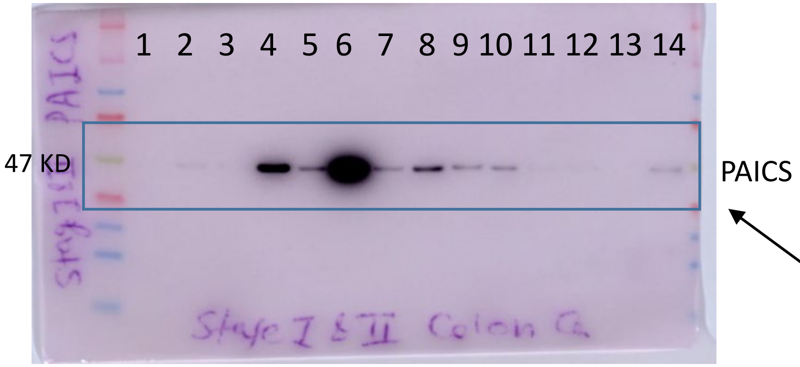

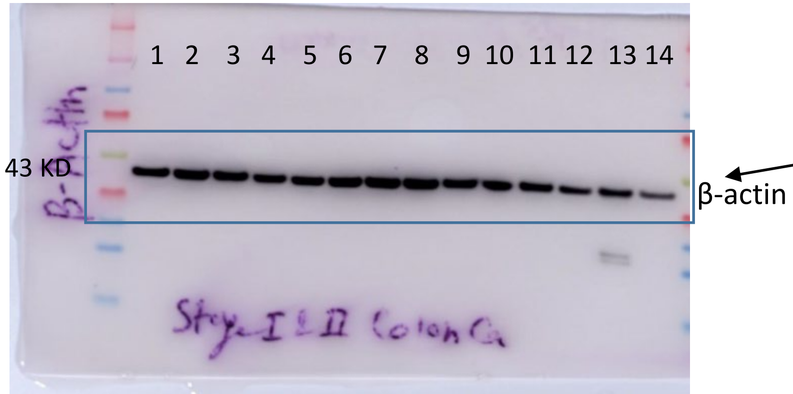

Figure provided in manuscript; Fig 2A

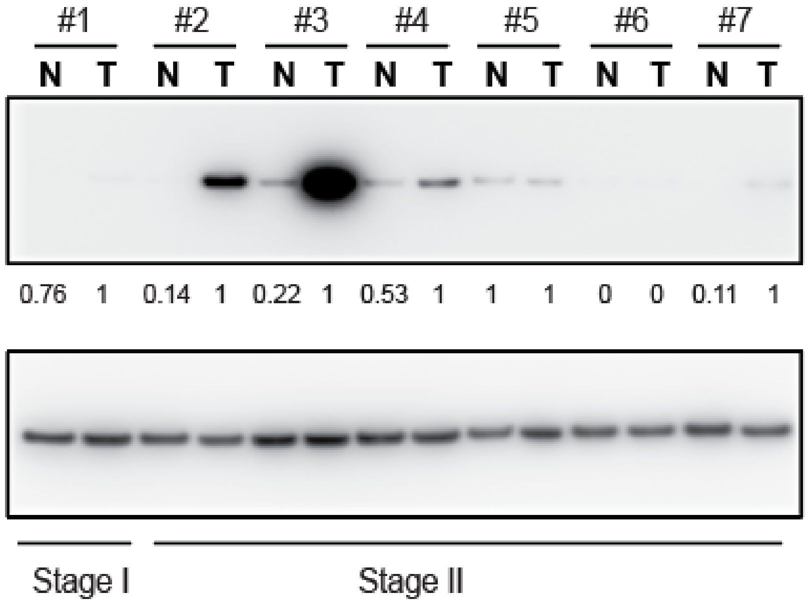

PAICS overexpression in colon cancer tissues, Fig 2A

STAGE III & IV

1. 29N STAGE III
2. 29T STAGE III
3. 70N STAGE III
4. 70T STAGE III
5. 79N STAGE III
6. 79N STAGE III
7. 330N STAGE III
8. 330T STAGE III
9. 1N STAGE IV
10. 1T STAGE IV
11. 98N STAGE IV
12. 98T STAGE IV
13. 126N STAGE IV
14. 126T STAGE IV

Raw blots; Fig 2A

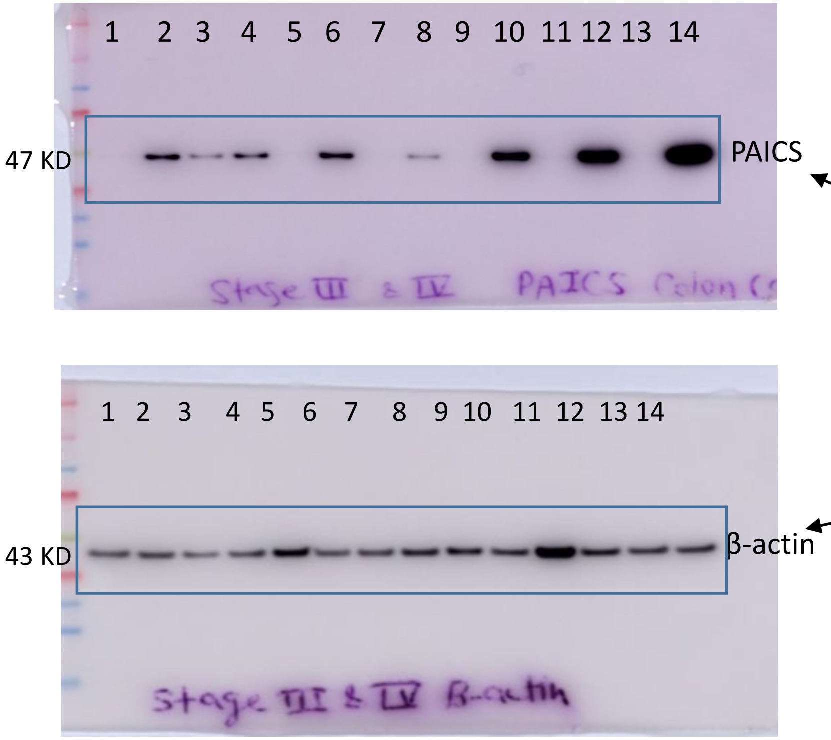

Figure provided in manuscript; Fig 2A

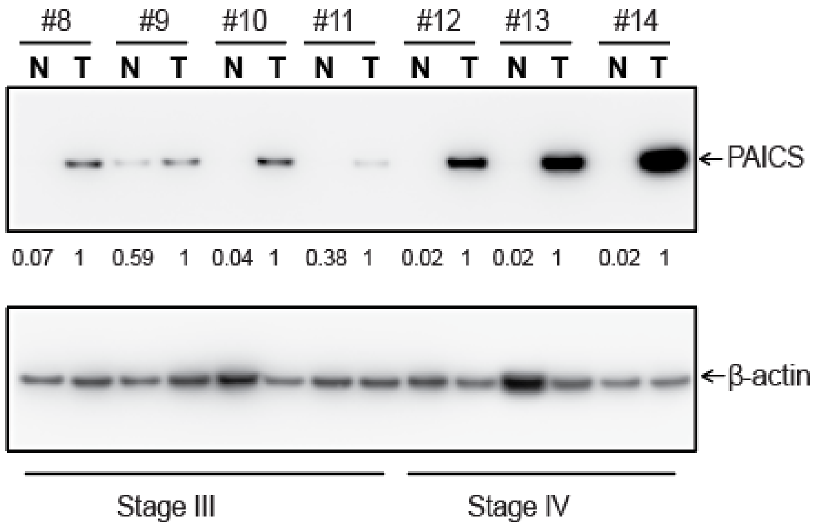

PAICS overexpression in colon cancer cells, Fig 2C

1. CRL1807
2. HCT116 wild
3. HCT116 p53 null
4. SW480

Raw blots; Fig 2C

Figure provided in manuscript; Fig 2C

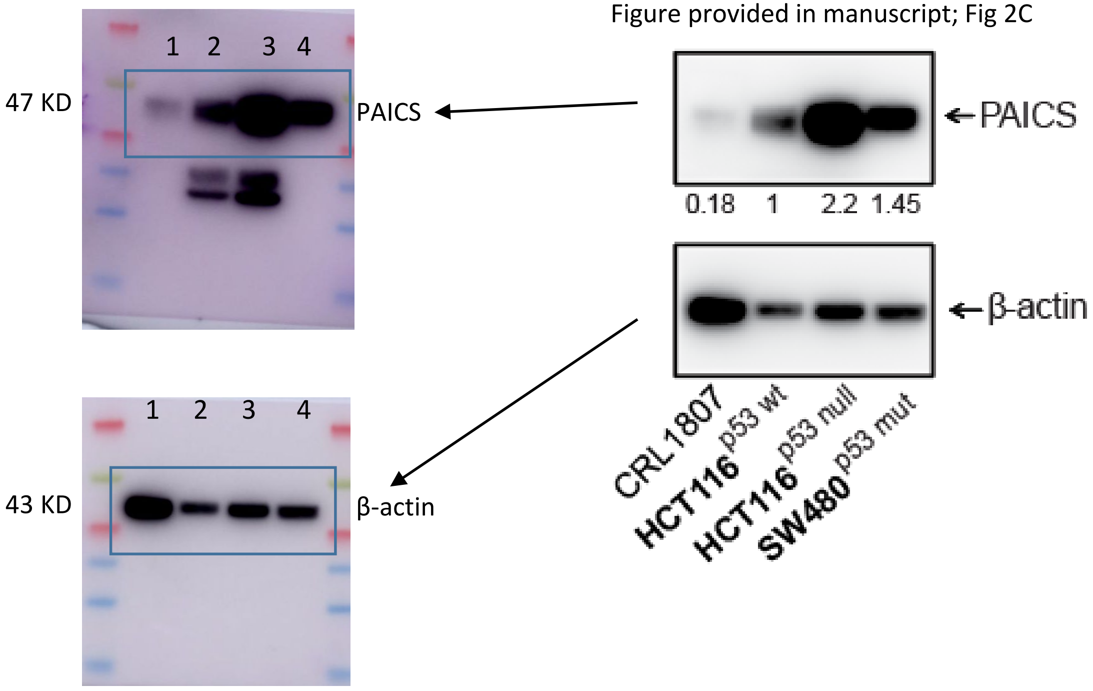

PAICS KD in colon cancer cells, Fig 3A

1. HCT116w SCR
2. HCT116w PAICS sh1
3. HCT116w PAICS sh2
4. HCT116p53- SCR
5. HCT116p53- PAICS sh1
6. HCT116p53- PAICS sh2

Raw blots for HCT116 p53 wild and p53 null cells; Fig 3A

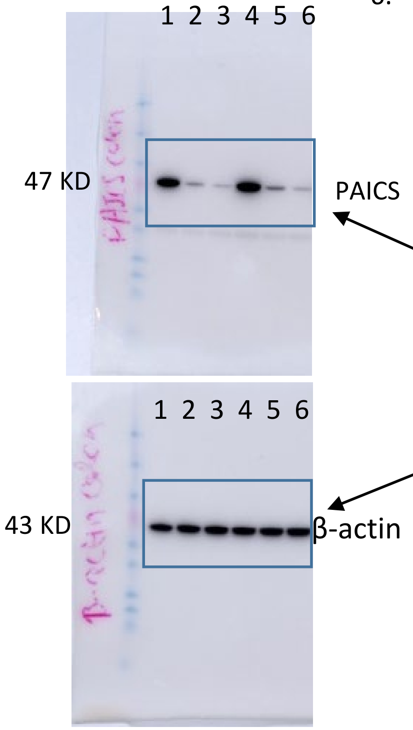

Figure provided in manuscript for HCT116 p53 wild and p53 null cells; Fig 3A

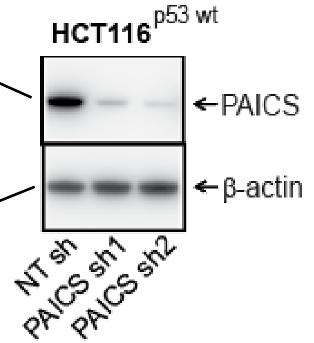

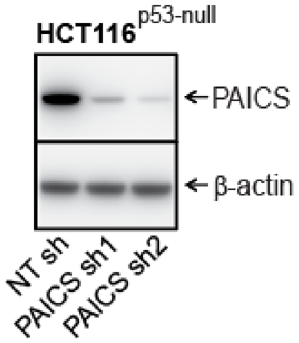

1. SW480 SCR
2. SW480 PAICS sh1
3. SW480 PAICS sh2

Raw blots for SW480; Fig 3A

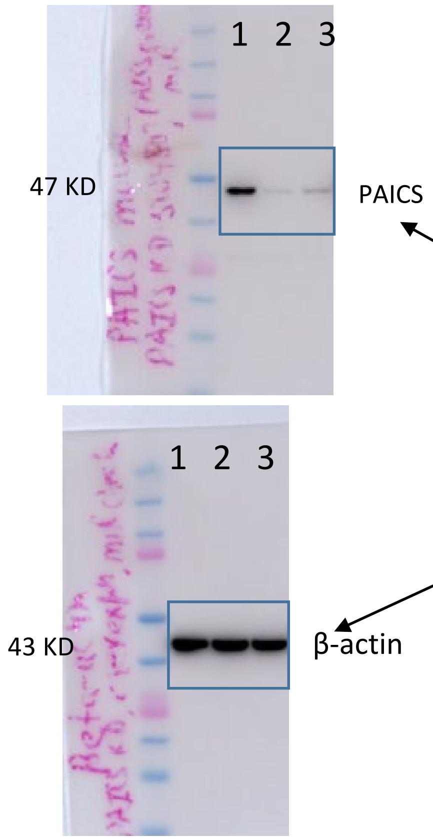

Figure provided in manuscript for SW480 cells; Fig 4A

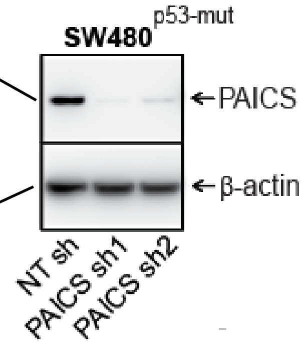

PAICS KD in HT29 LUC cells for metastasis, Fig 4A

1. HT29 LUC SCR
2. HT29 LUC PAICS sh1

Raw blots; Fig 4A

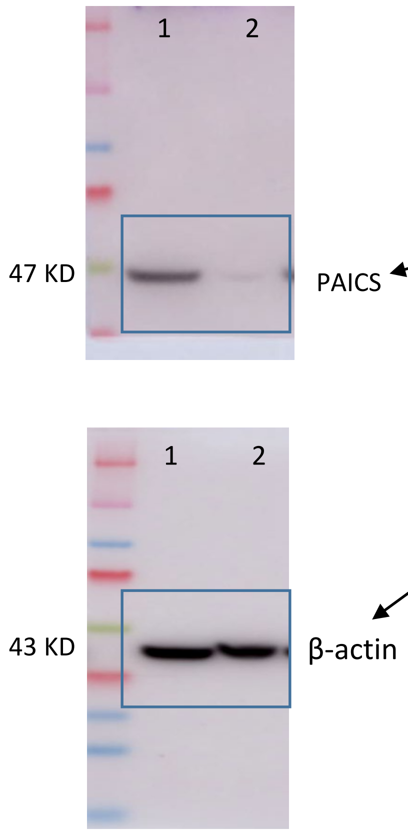

Figure provided in manuscript; Fig 4A

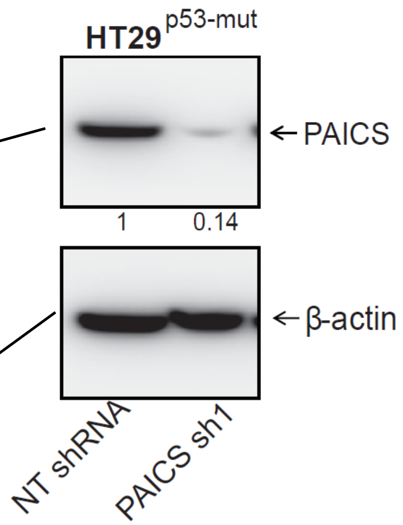

E cadherin levels after PAICS KD, Fig 6B

1. HCT116w SCR
2. HCT116w PAICS sh1
3. HCT116w PAICS sh2
4. HCT116p53-null SCR
5. HCT116p53-null PAICS sh1
6. HCT116p53-null PAICS sh2
7. SW480 SCR
8. SW480 PAICS sh1
9. SW480 PAICS sh2

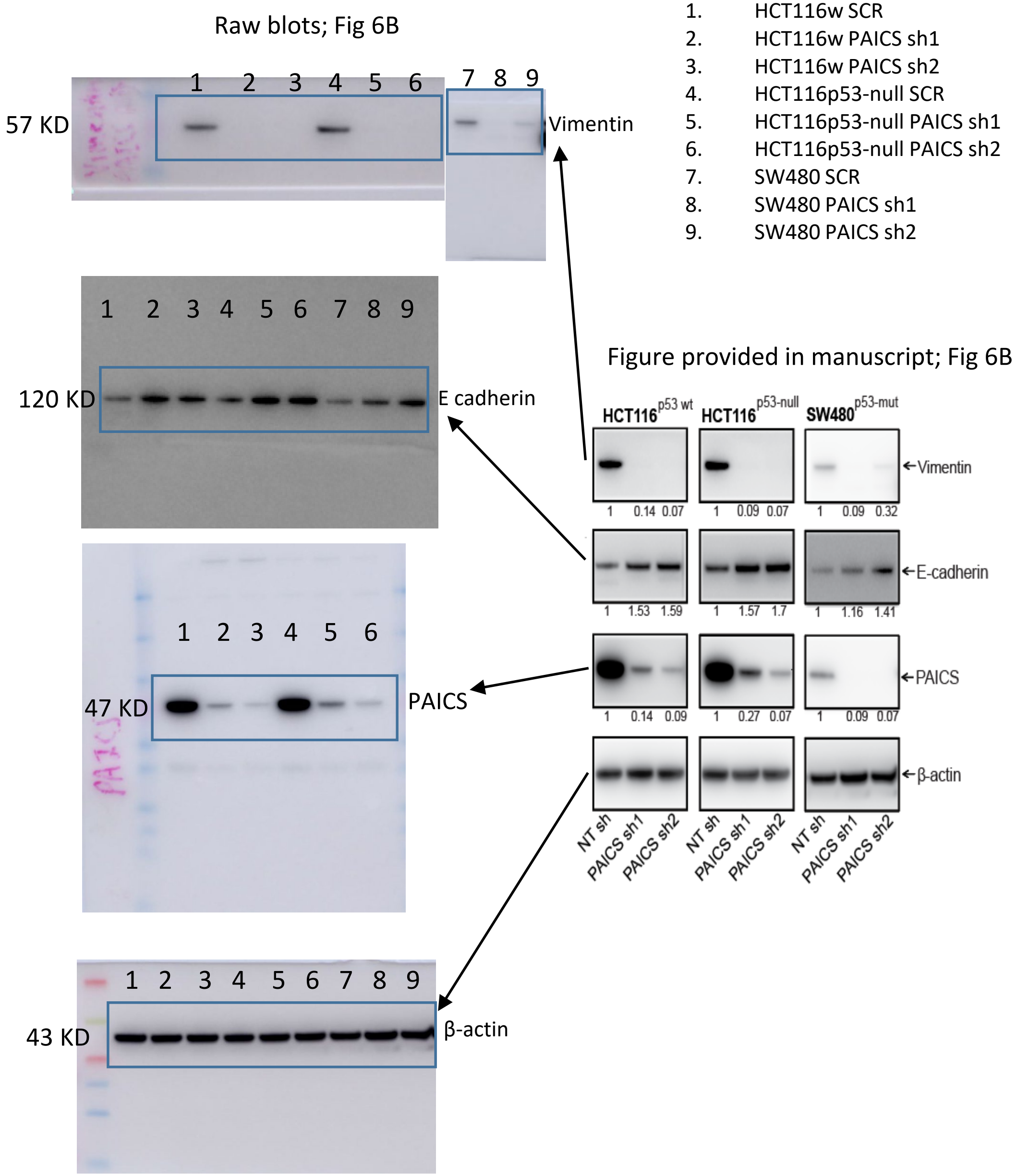

Xenograft after PAICS KD, Fig 6D

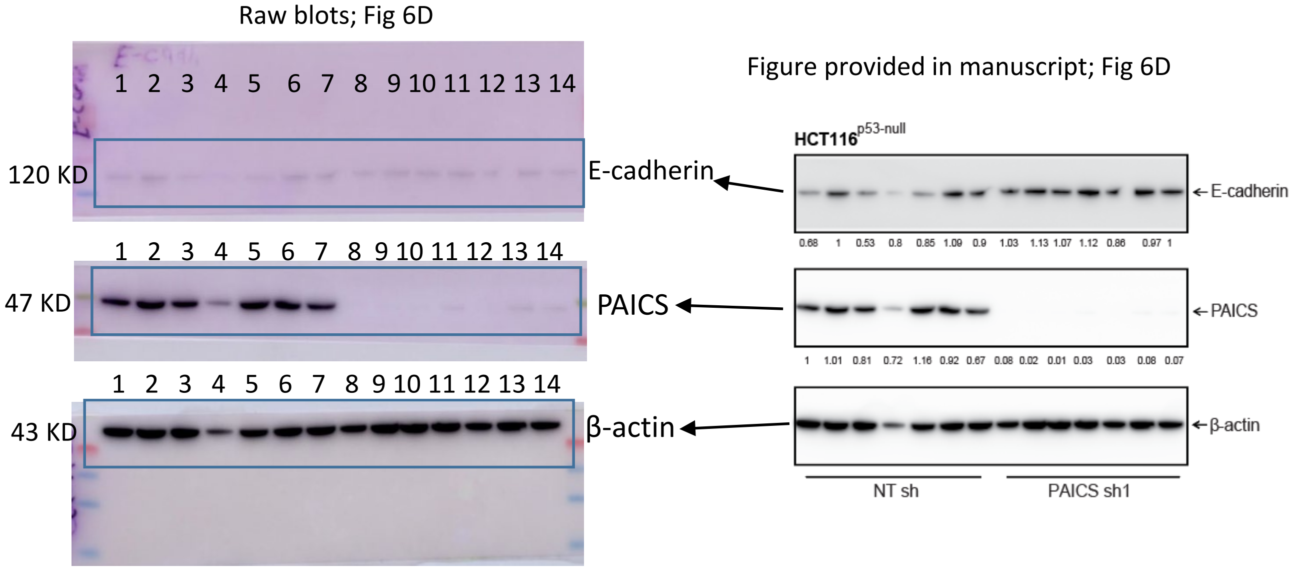

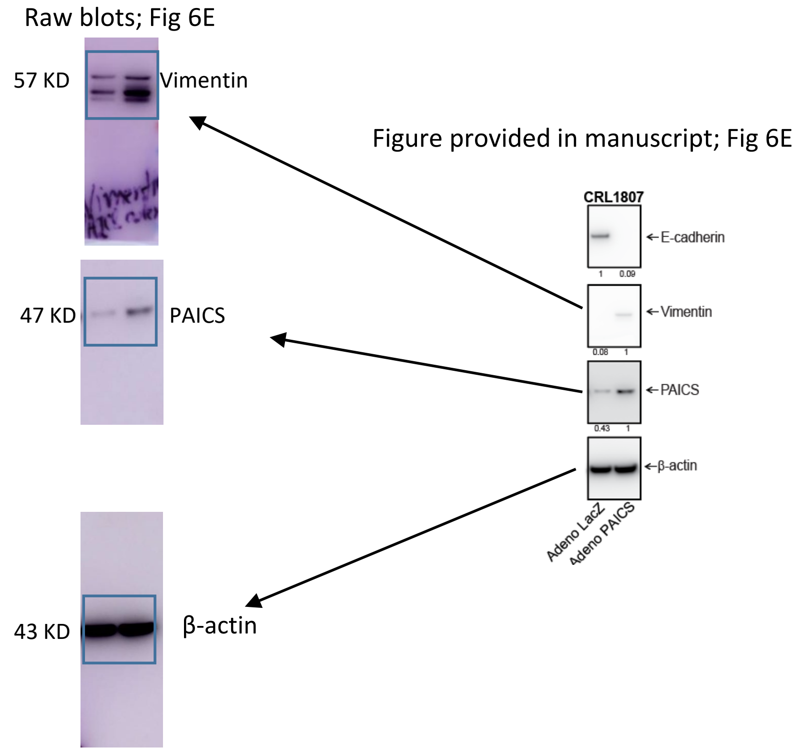

miR128 regulates PAICS in CRC; Fig 7C

1. M + HCT116w NT miR
2. HCT116w miR128a
3. HCT116w miR141
4. HCT116w miR146

Raw blots; Fig 7C

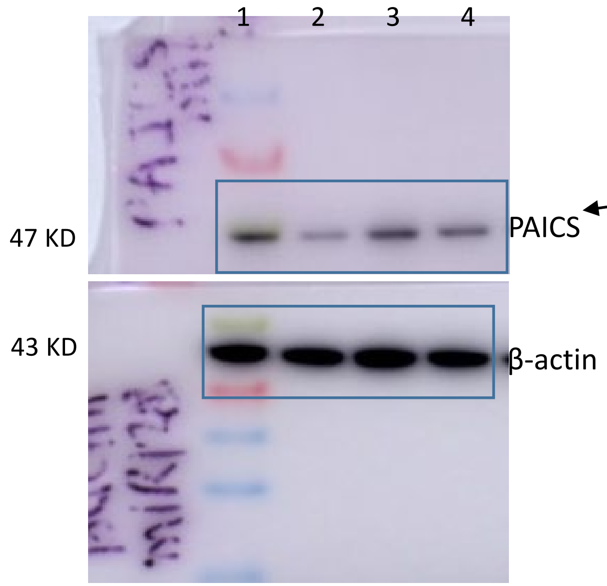

Figure provided in manuscript; Fig 7C

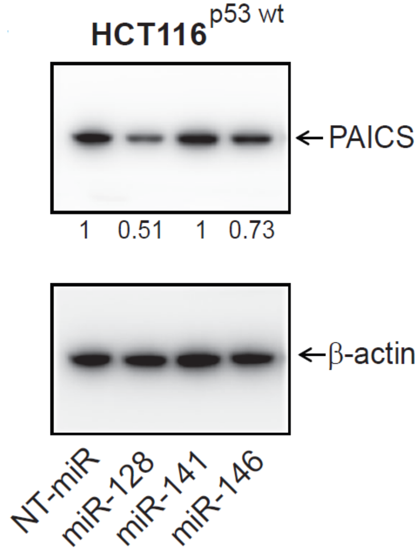

Raw blots; Fig 7G

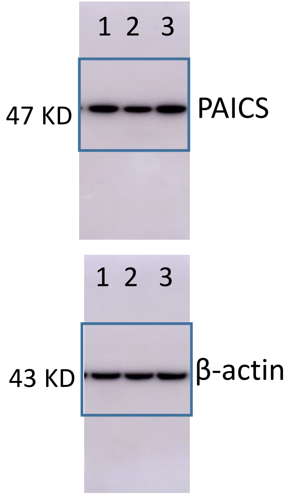

Figure provided in manuscript; Fig 7C

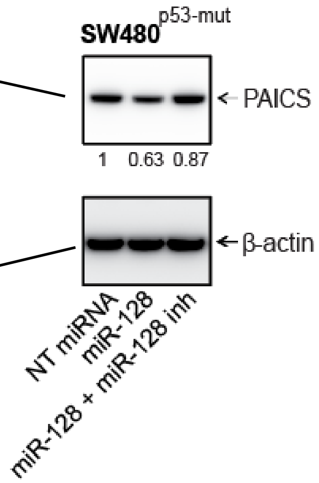

c-MYC regulates PAICS in CRC; Fig 8

1. CRL1807 adeno LacZ
2. CRL1807 adeno PAICS

Raw blots; Fig 8B

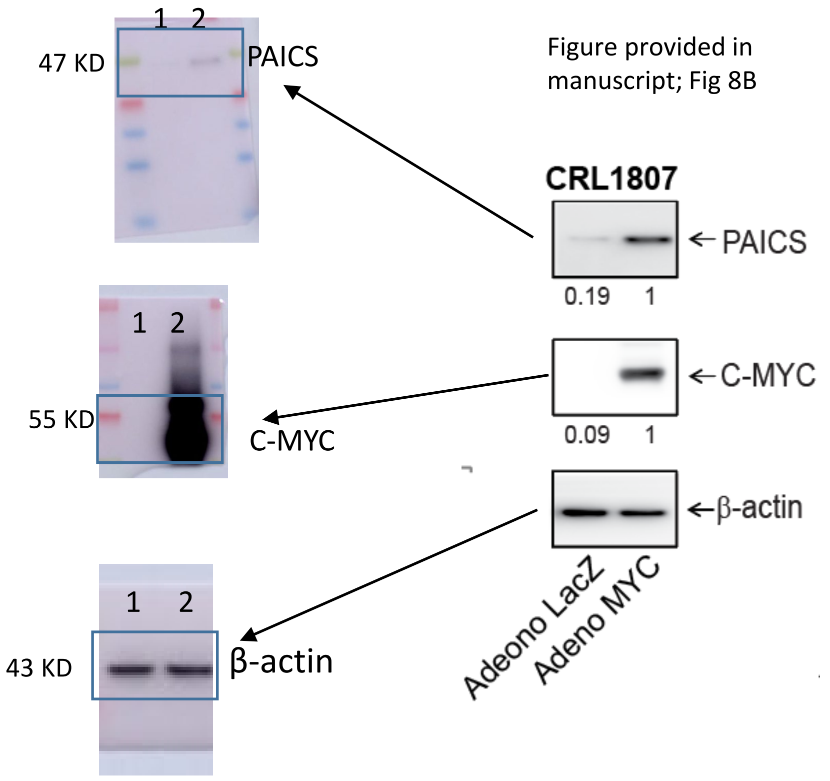

Raw blots; Fig 8C

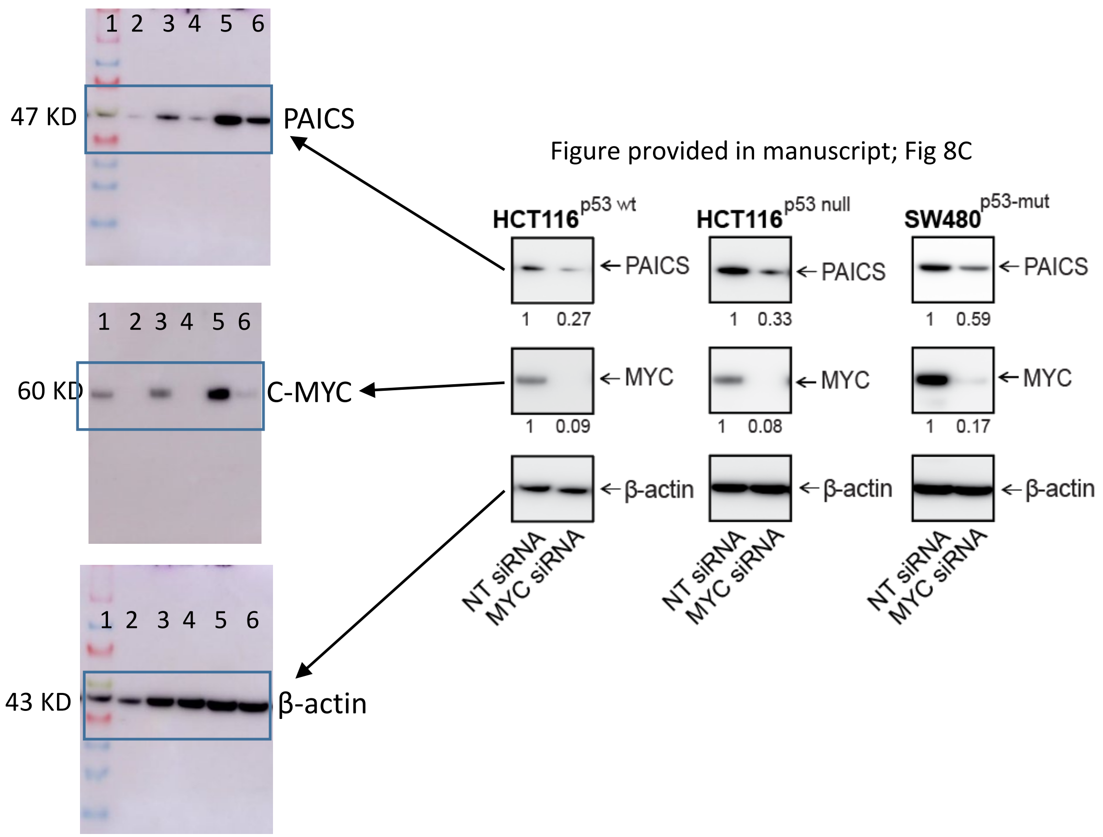

c-MYC regulates PAICS in CRC; Fig 8

Raw blots for HCT116 p53 wild and p53 null cells; Fig 8D

1. HCT116 w DMSO
2. HCT116w 2.5 uM JQ1
3. HCT116w 5 uM JQ1
4. HCT116 p53 null DMSO
5. HCT116 p53 null 2.5 uM JQ1
6. HCT116 p53 null 5 uM JQ1

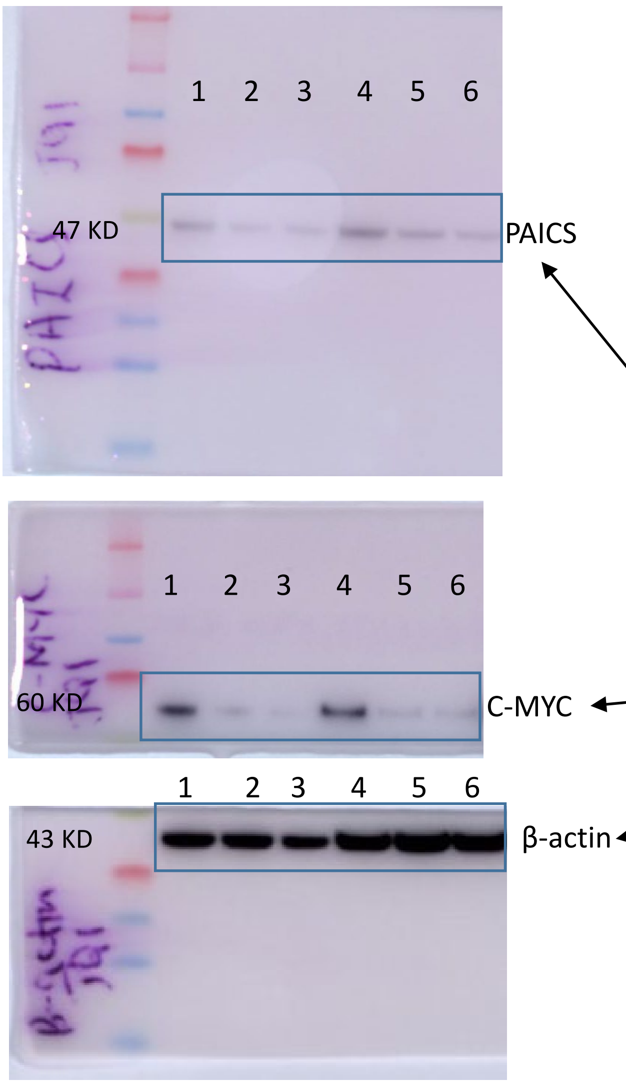

Figure provided in manuscript; Fig 8D

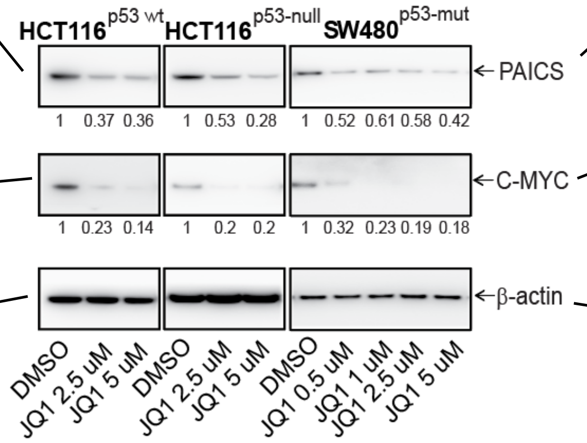

1. SW480 DMSO
2. SW480 JQ1 500nM
3. SW480 JQ1 1uM
4. SW480 JQ1 2.5 uM
5. SW480 JQ1 5 uM

Raw blots for SW480; Fig 8D

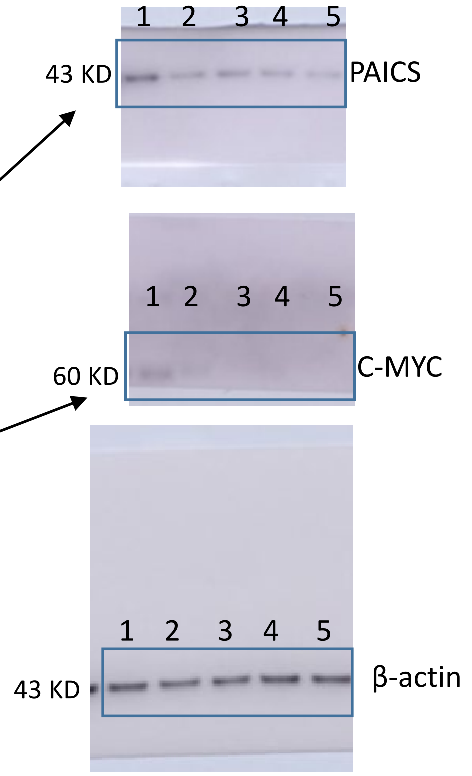

Supplement: Supplementary file 1 [file cancers-12-00772-s001.pdf]
